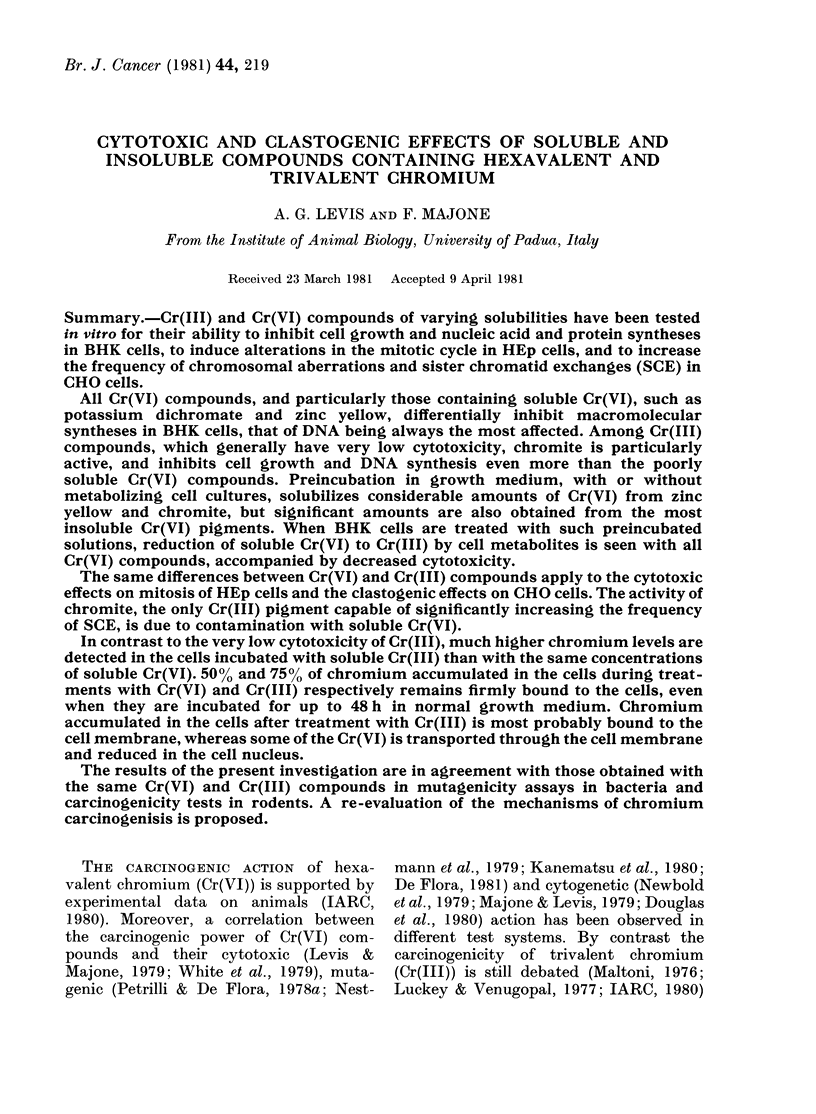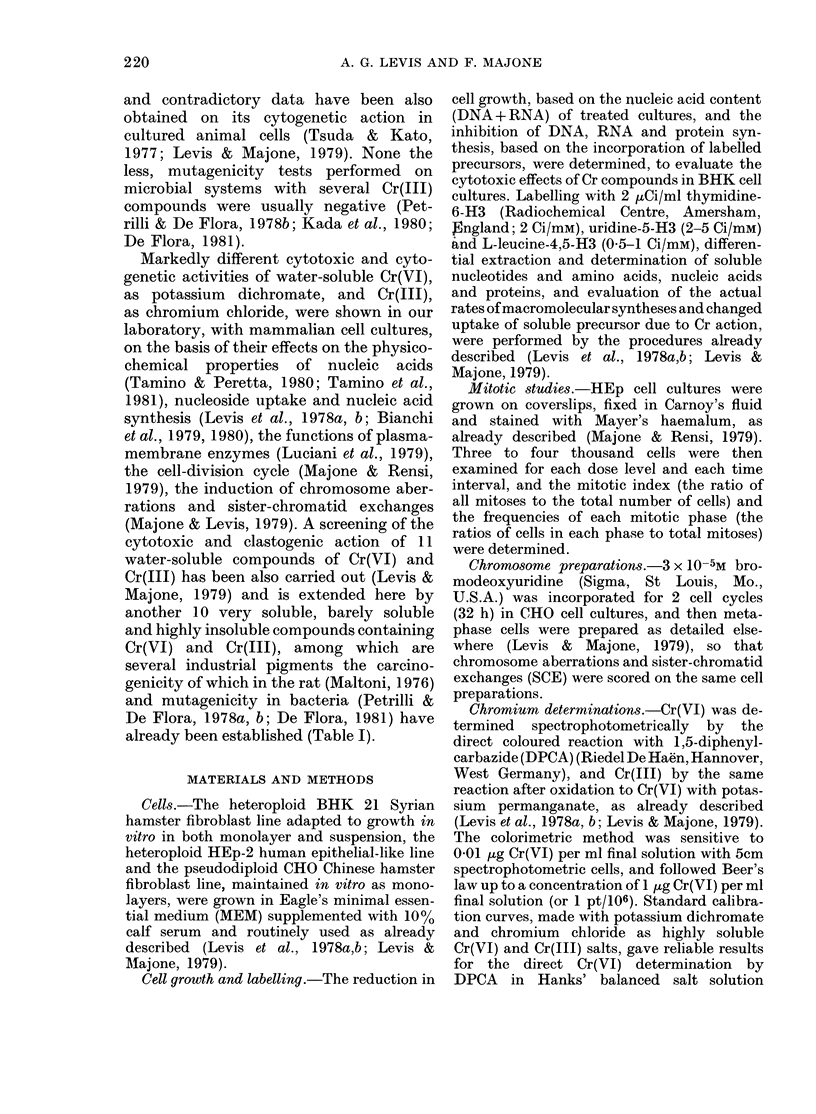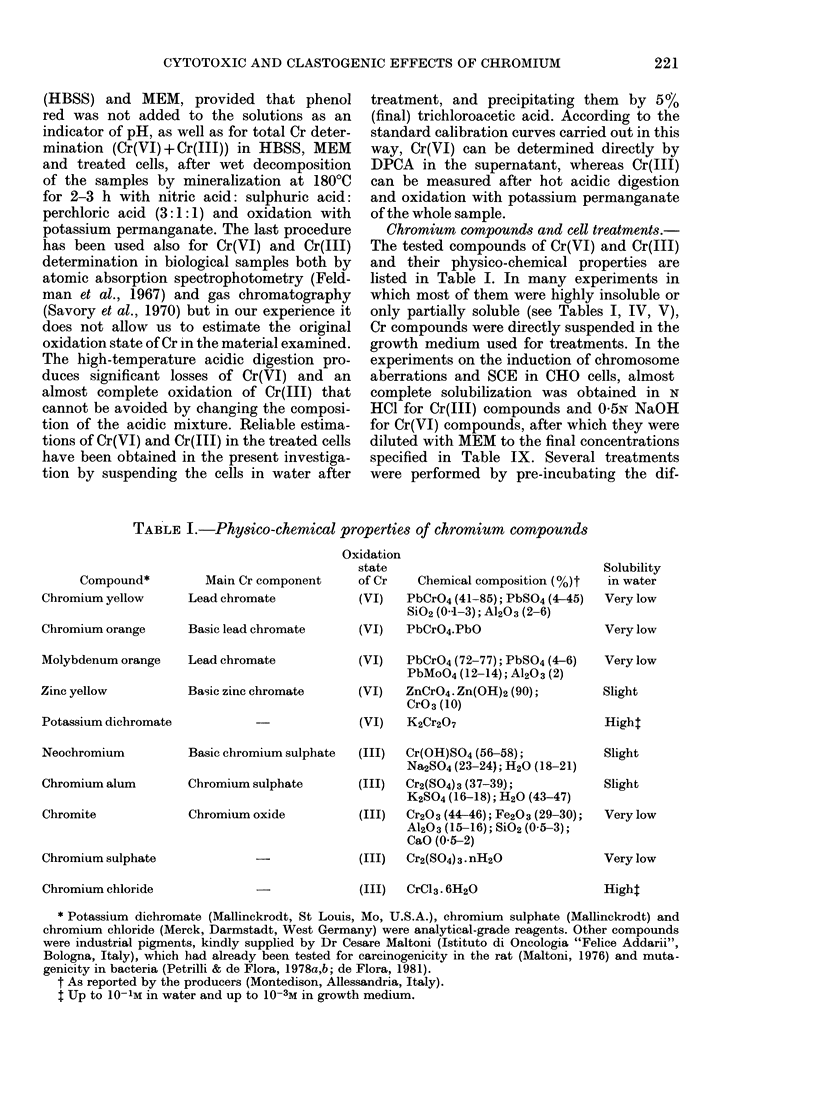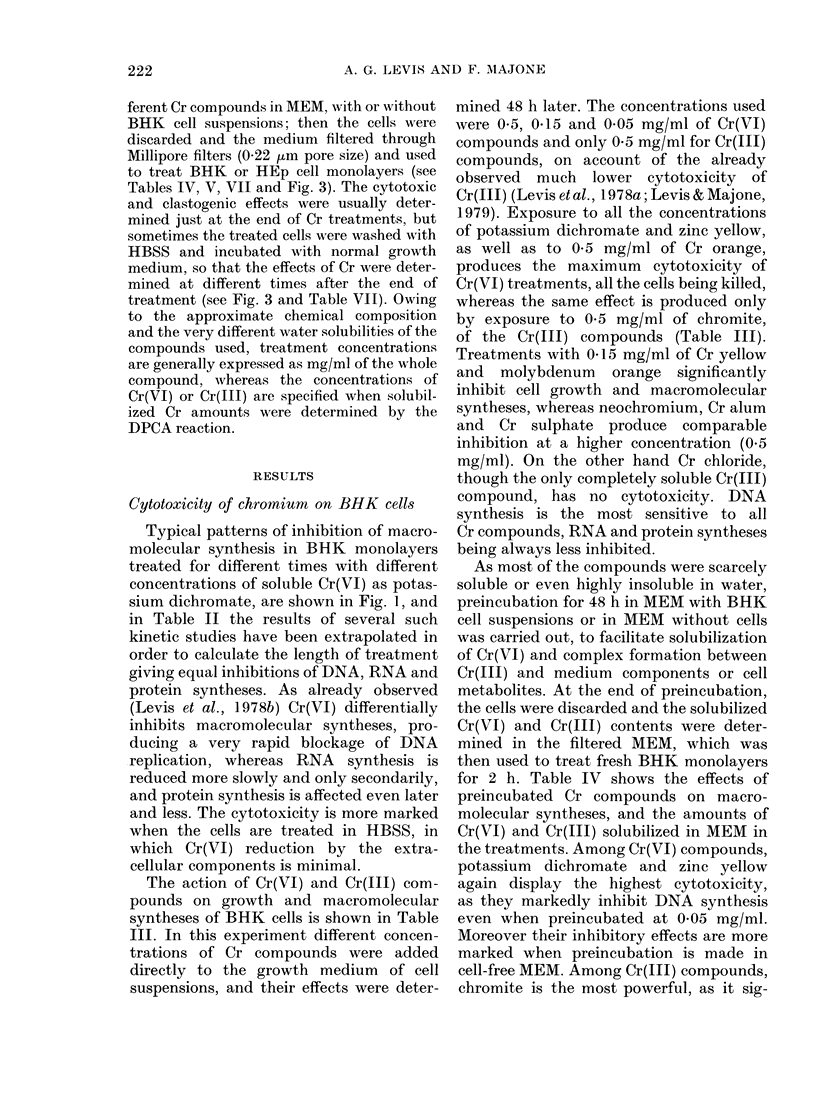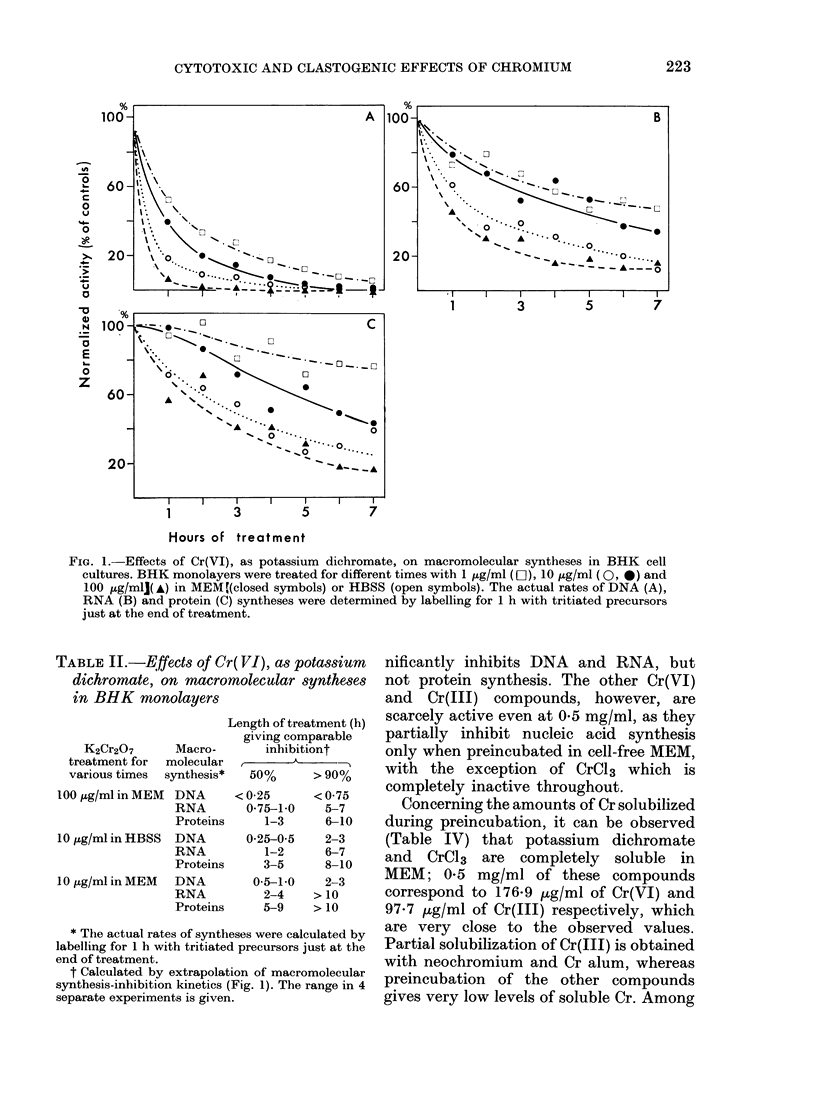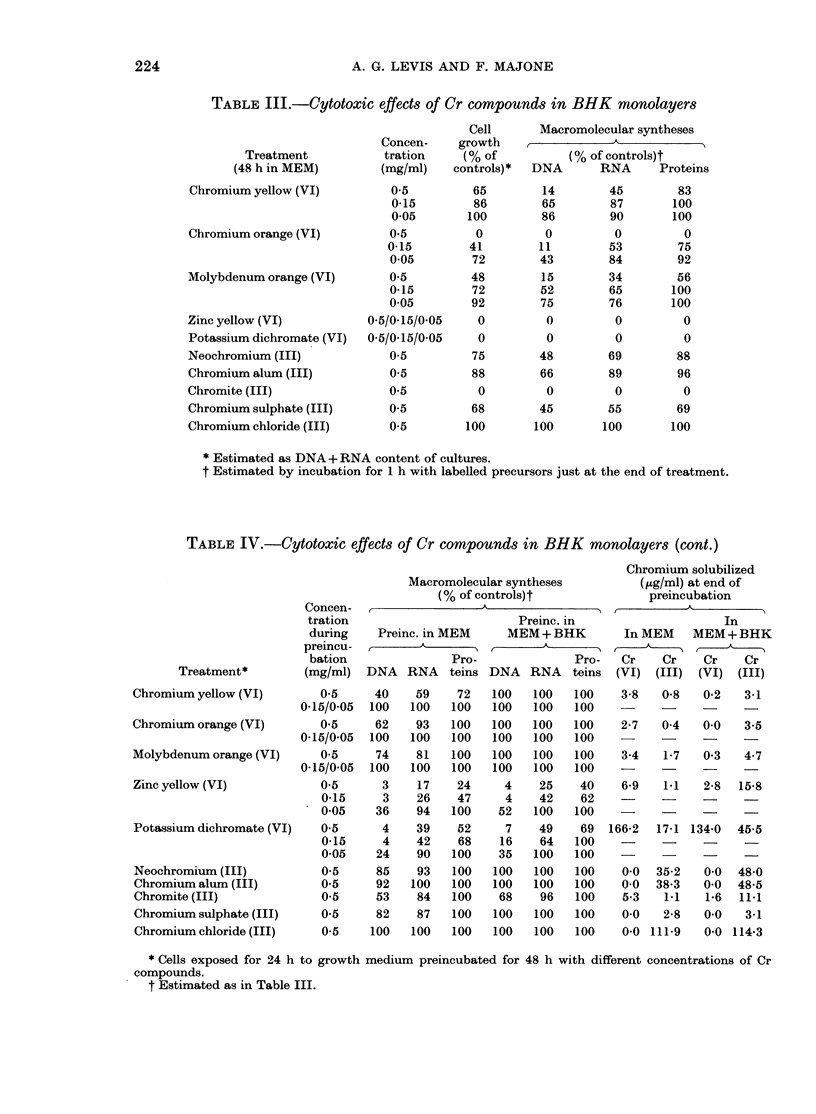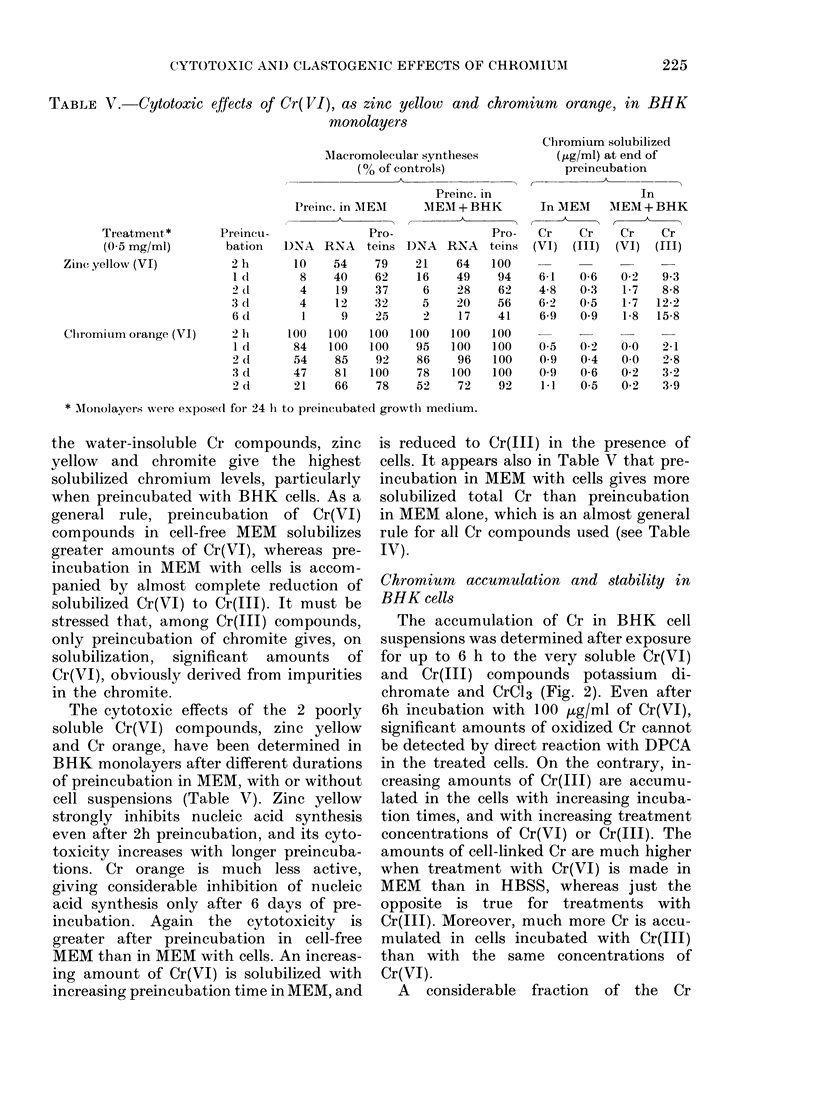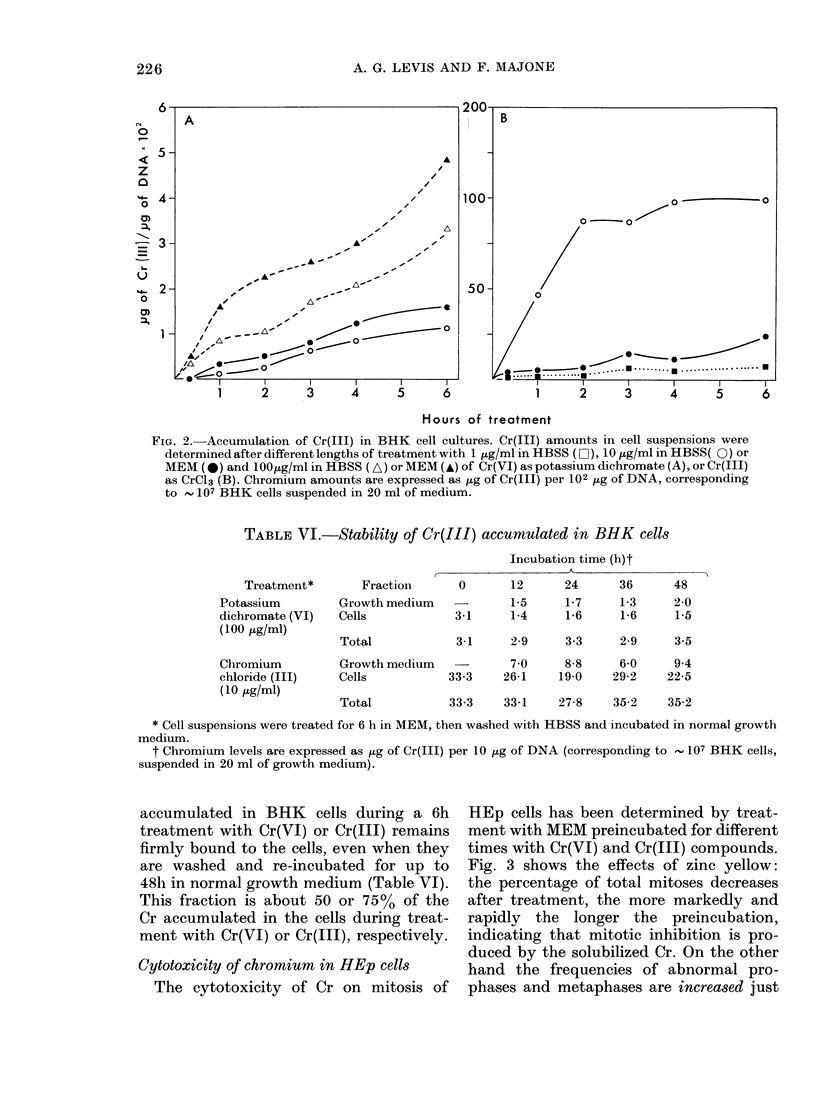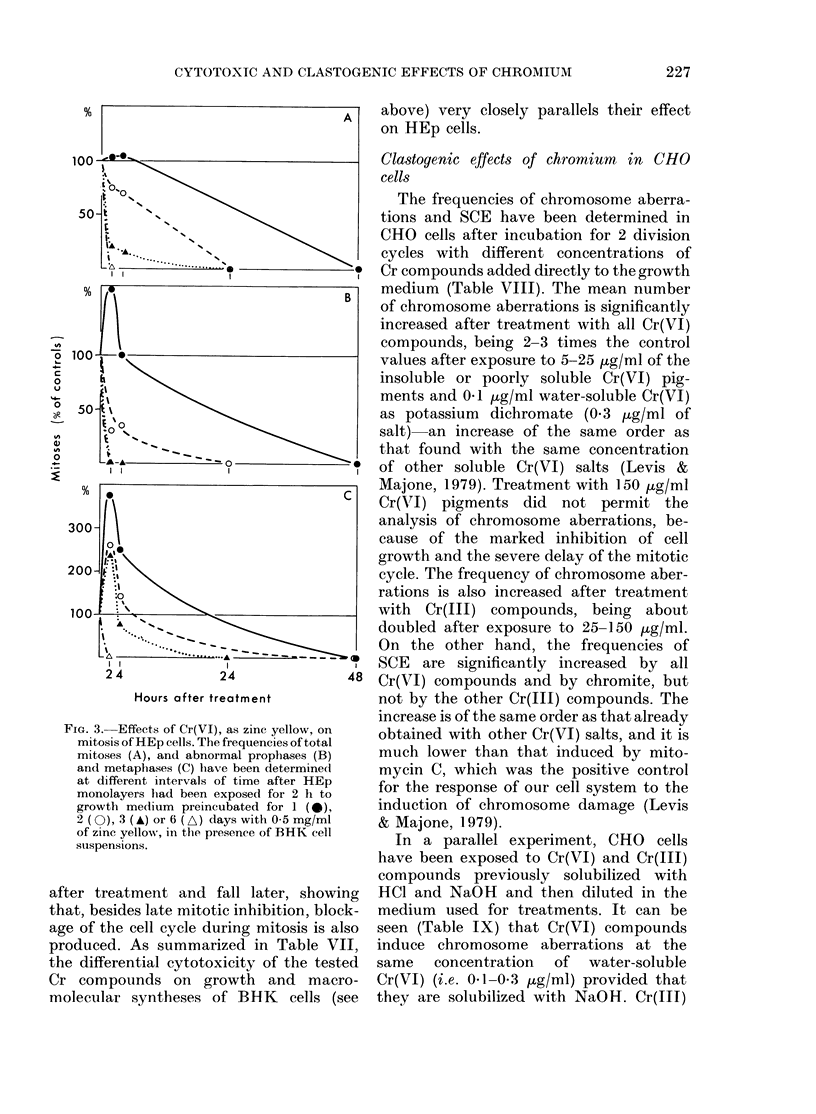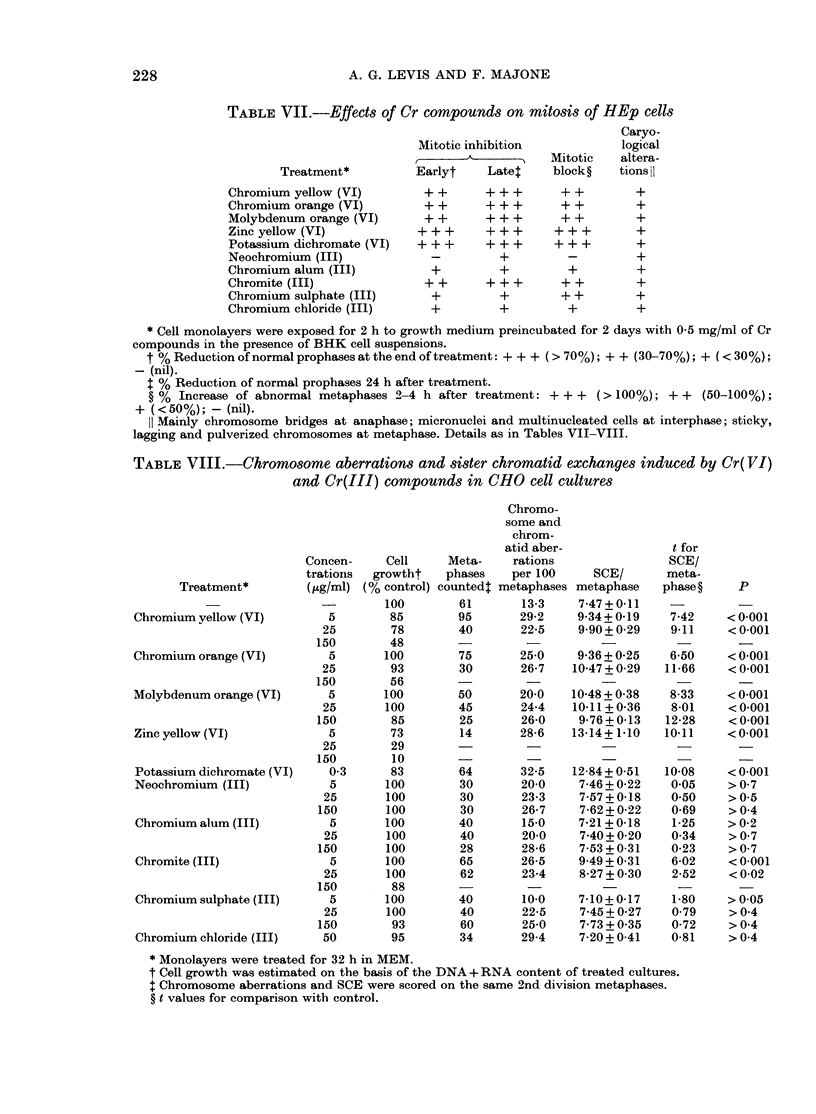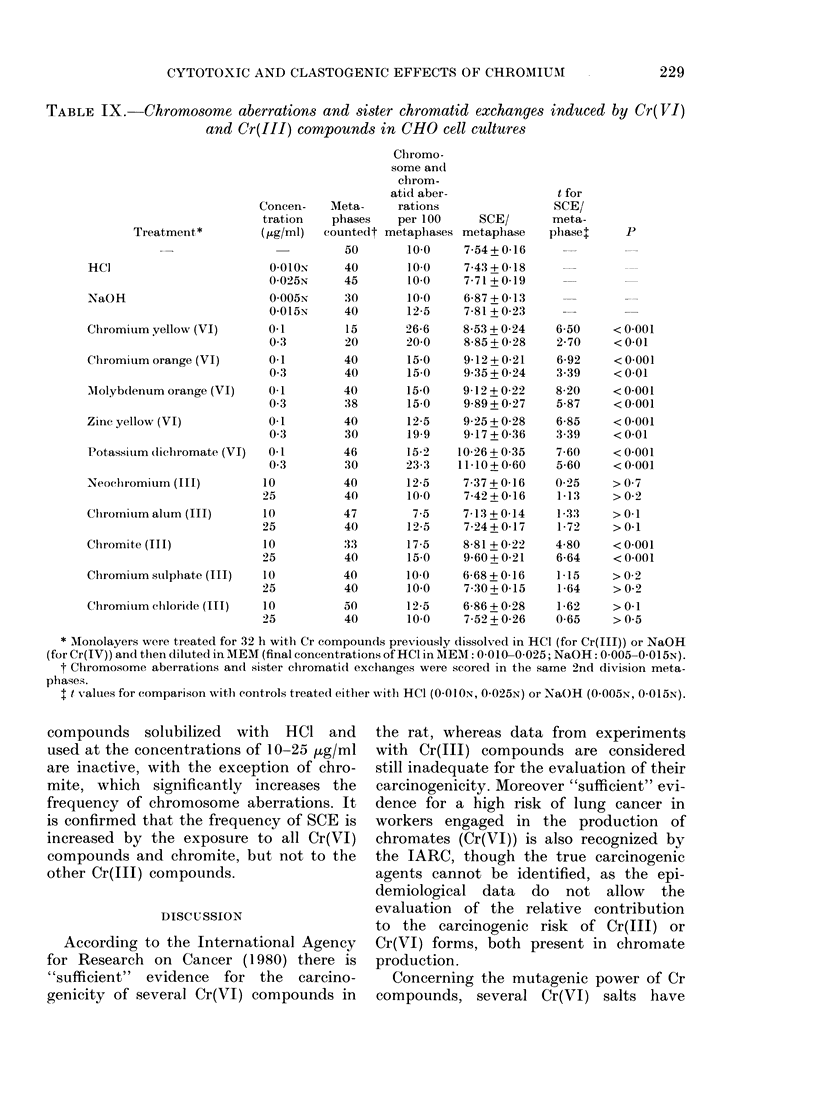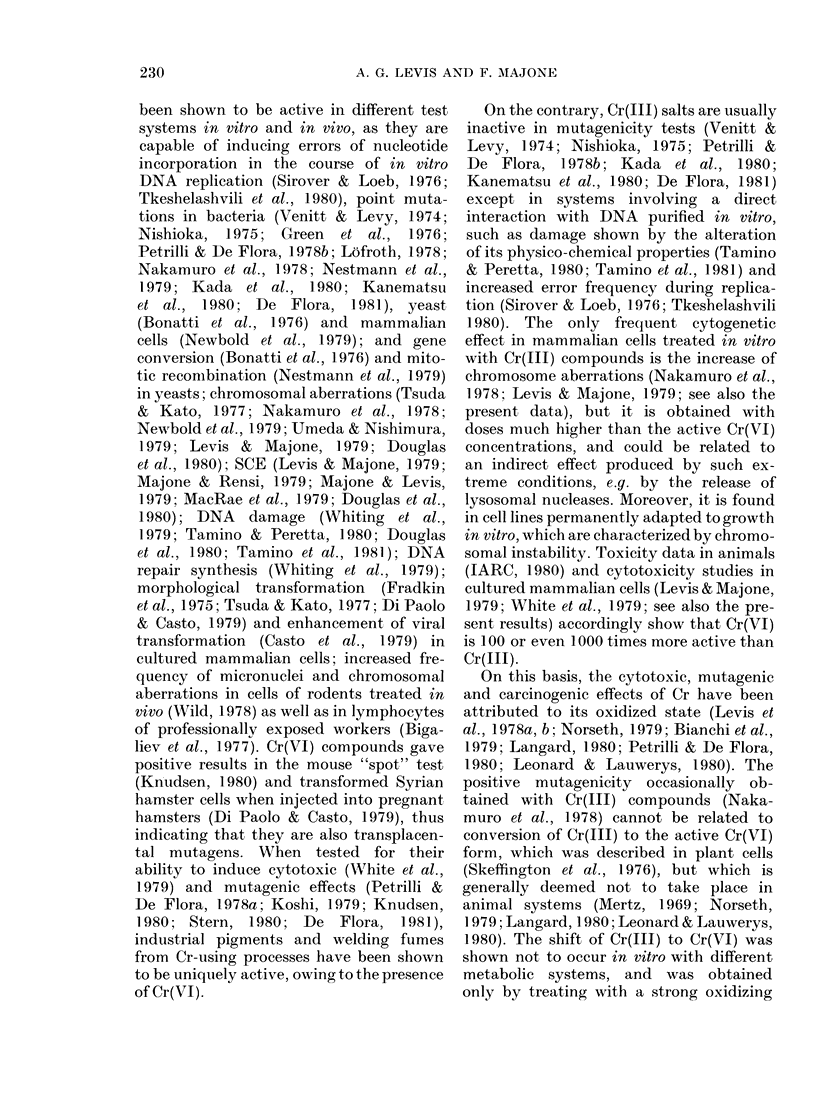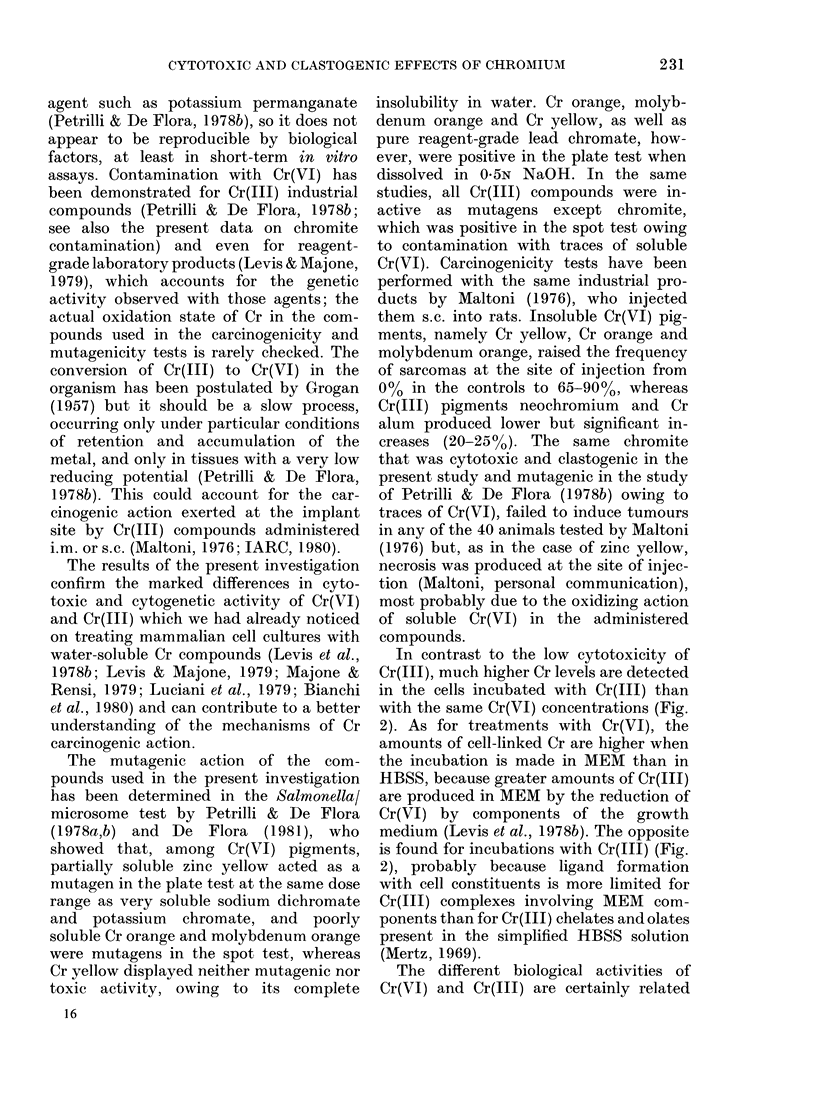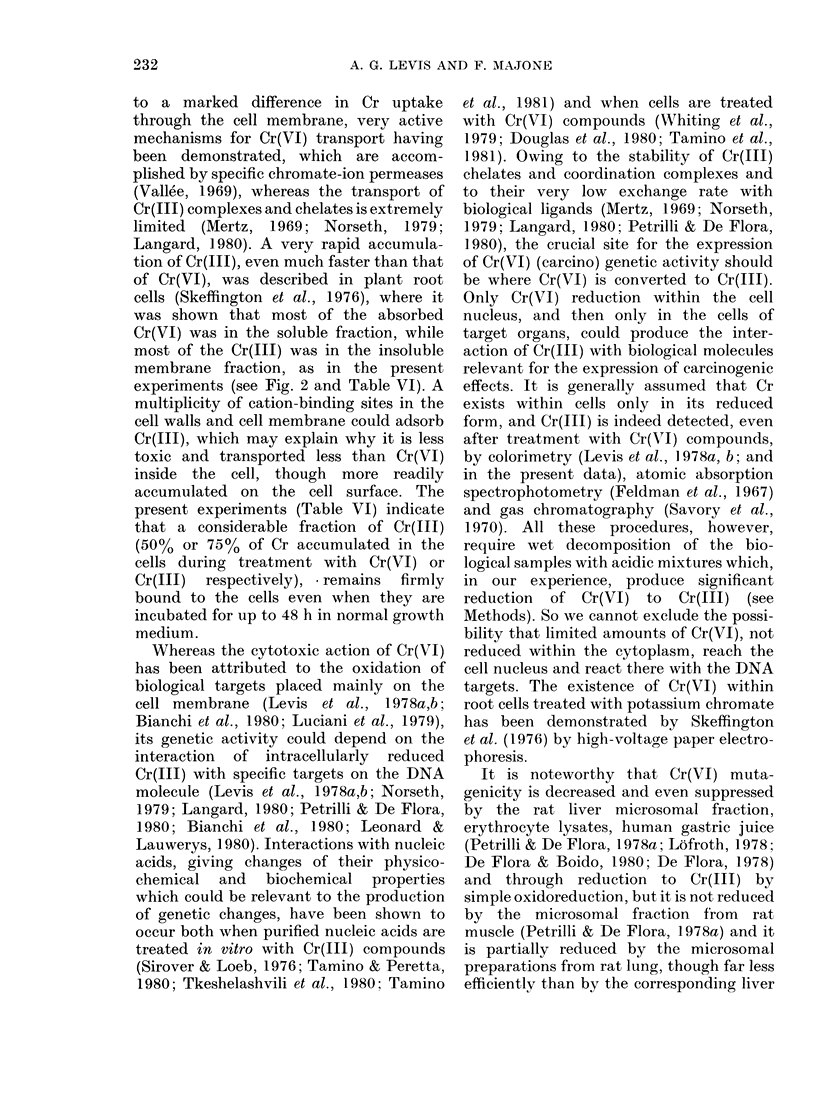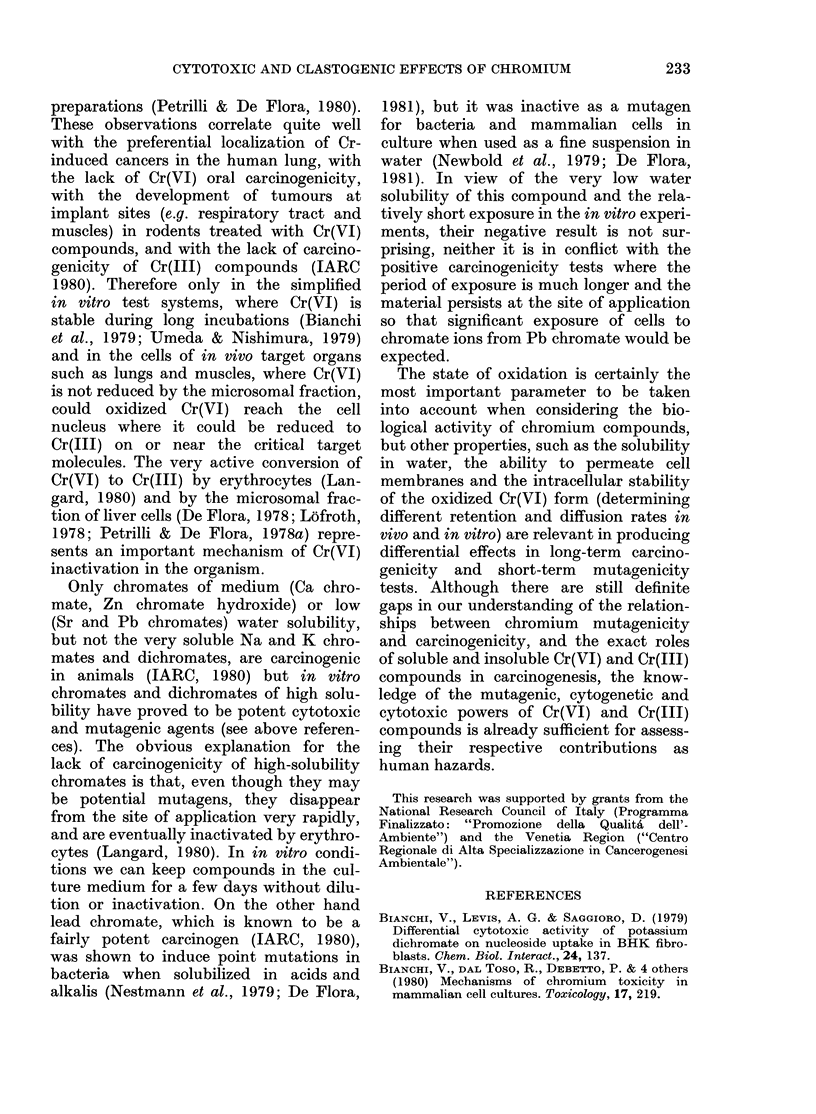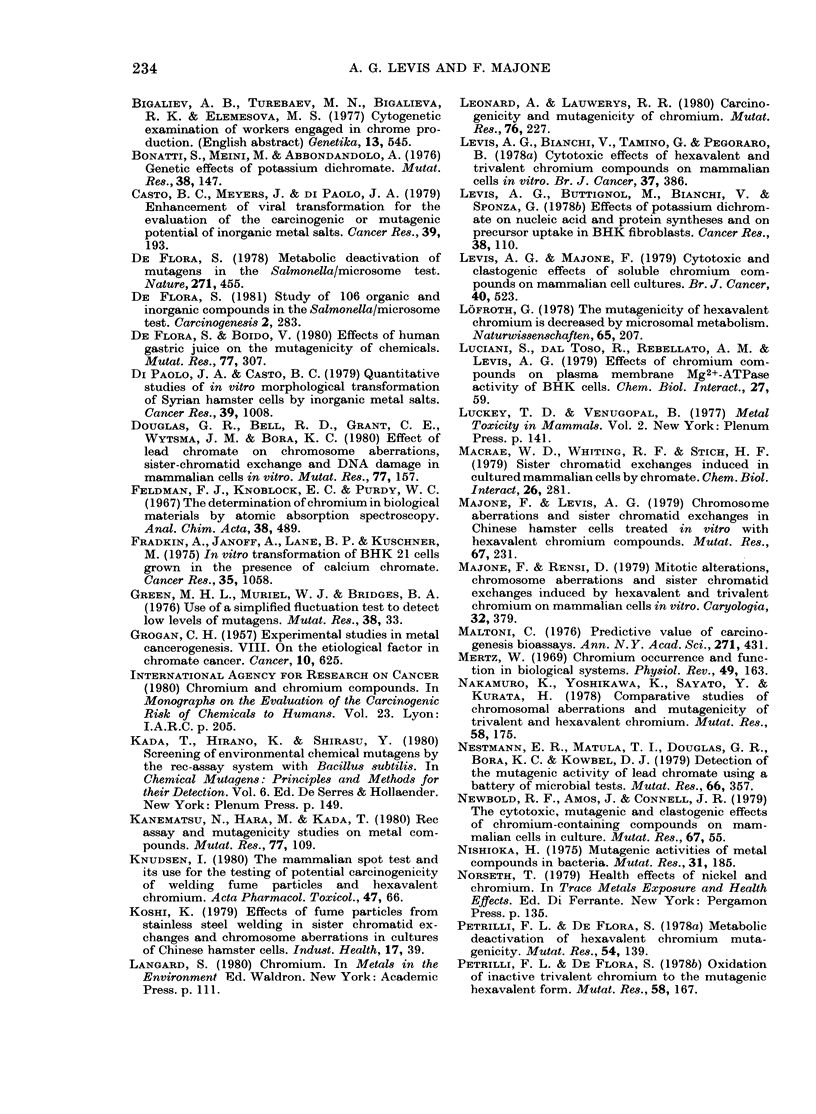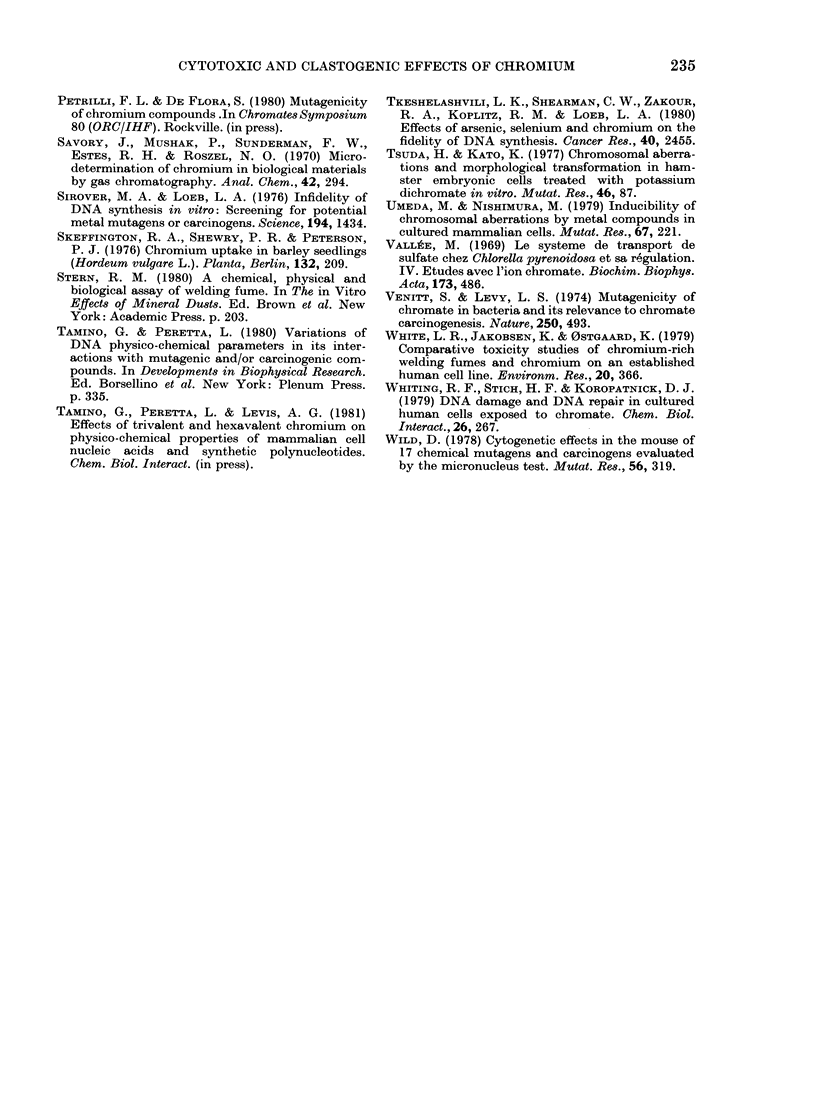# Cytotoxic and clastogenic effects of soluble and insoluble compounds containing hexavalent and trivalent chromium.

**DOI:** 10.1038/bjc.1981.173

**Published:** 1981-08

**Authors:** A. G. Levis, F. Majone

## Abstract

Cr(III) and Cr(VI) compounds of varying solubilities have been tested in vitro for their ability to inhibit cell growth and nucleic acid and protein syntheses in BHK cells, to induce alterations in the mitotic cycle in HEp cells, and to increase the frequency of chromosomal aberrations and sister chromatid exchanges (SCE) in CHO cells. All Cr(VI) compounds, and particularly those containing soluble Cr(VI), such as potassium dichromate and zinc yellow, differentially inhibit macromolecular syntheses in BKH cells, that of DNA being always the most affected. Among Cr(III) compounds, which generally have very low cytotoxicity, chromite is particularly active, and inhibits cell growth and DNA synthesis even more than the poorly soluble Cr(VI) compounds. Preincubation in growth medium, with or without metabolizing cell cultures, solubilizes considerable amounts of Cr(VI) from zinc yellow and chromite, but significant amounts are also obtained from the most insoluble Cr(VI) pigments. When BHK cells are treated with such preincubated solutions, reduction of soluble Cr(VI) to Cr(III) by cell metabolites is seen with all Cr(VI) compounds, accompanied by decreased cytotoxicity. The same differences between Cr(VI) and Cr(III) compounds apply to the cytotoxic effects on mitosis of HEp cells and the clastogenic effects on CHO cells. The activity of chromite, the only Cr(III) pigment capable of significantly increasing the frequency of SCE, is due to contamination with soluble Cr(VI). In contrast to the very low cytotoxicity of Cr(III), much higher chromium levels are detected in the cells incubated with soluble Cr(III) than with the same concentrations of soluble Cr(VI). 50% and 75% of chromium accumulated in the cells during treatments with Cr(VI) and Cr(III) respectively remains firmly bound to the cells, even when they are incubated for up to 48 h in normal growth medium. Chromium accumulated in the cells after treatment with Cr(III) is most probably bound to the cell membrane, whereas some of the Cr(VI) is transported through the cell membrane and reduced in the cell nucleus. The results of the present investigation are in agreement with those obtained with the same Cr(VI) and Cr(III) compounds in mutagenicity assays in bacteria and carcinogenicity tests in rodents. A re-evaluation of the mechanisms of chromium carcinogenisis is proposed.


*Br. J. Cancer* (1981) **44**, 219

### CYTOTOXIC AND CLASTOGENIC EFFECTS OF SOLUBLE AND INSOLUBLE COMPOUNDS CONTAINING HEXAVALENT AND TRIVALENT CHROMIUM

A. G. LEVIS AND F. MAJONE

*From the Institute of Animal Biology, University of Padua, Italy*

Received 23 March 1981 Accepted 9 April 1981

**Summary.—Cr(III) and Cr(VI) compounds of varying solubilities have been tested *in vitro* for their ability to inhibit cell growth and nucleic acid and protein syntheses in BHK cells, to induce alterations in the mitotic cycle in HEp cells, and to increase the frequency of chromosomal aberrations and sister chromatid exchanges (SCE) in CHO cells.**

**All Cr(VI) compounds, and particularly those containing soluble Cr(VI), such as potassium dichromate and zinc yellow, differentially inhibit macromolecular syntheses in BHK cells, that of DNA being always the most affected. Among Cr(III) compounds, which generally have very low cytotoxicity, chromite is particularly active, and inhibits cell growth and DNA synthesis even more than the poorly soluble Cr(VI) compounds. Preincubation in growth medium, with or without metabolizing cell cultures, solubilizes considerable amounts of Cr(VI) from zinc yellow and chromite, but significant amounts are also obtained from the most insoluble Cr(VI) pigments. When BHK cells are treated with such preincubated solutions, reduction of soluble Cr(VI) to Cr(III) by cell metabolites is seen with all Cr(VI) compounds, accompanied by decreased cytotoxicity.**

**The same differences between Cr(VI) and Cr(III) compounds apply to the cytotoxic effects on mitosis of HEp cells and the clastogenic effects on CHO cells. The activity of chromite, the only Cr(III) pigment capable of significantly increasing the frequency of SCE, is due to contamination with soluble Cr(VI).**

**In contrast to the very low cytotoxicity of Cr(III), much higher chromium levels are detected in the cells incubated with soluble Cr(III) than with the same concentrations of soluble Cr(VI). 50% and 75% of chromium accumulated in the cells during treatments with Cr(VI) and Cr(III) respectively remains firmly bound to the cells, even when they are incubated for up to 48 h in normal growth medium. Chromium accumulated in the cells after treatment with Cr(III) is most probably bound to the cell membrane, whereas some of the Cr(VI) is transported through the cell membrane and reduced in the cell nucleus.**

**The results of the present investigation are in agreement with those obtained with the same Cr(VI) and Cr(III) compounds in mutagenicity assays in bacteria and carcinogenicity tests in rodents. A re-evaluation of the mechanisms of chromium carcinogenisis is proposed.**

THE CARCINOGENIC ACTION of hexavalent chromium (Cr(VI)) is supported by experimental data on animals (IARC, 1980). Moreover, a correlation between the carcinogenic power of Cr(VI) compounds and their cytotoxic (Levis & Majone, 1979; White *et al.*, 1979), mutagenic (Petrilli & De Flora, 1978*a*; Nestmann *et al.*, 1979; Kanematsu *et al.*, 1980; De Flora, 1981) and cytogenetic (Newbold *et al.*, 1979; Majone & Levis, 1979; Douglas *et al.*, 1980) action has been observed in different test systems. By contrast the carcinogenicity of trivalent chromium (Cr(III)) is still debated (Maltoni, 1976; Luckey & Venugopal, 1977; IARC, 1980)

and contradictory data have been also obtained on its cytogenetic action in cultured animal cells (Tsuda & Kato, 1977; Levis & Majone, 1979). None the less, mutagenicity tests performed on microbial systems with several Cr(III) compounds were usually negative (Petrilli & De Flora, 1978*b*; Kada *et al.*, 1980; De Flora, 1981).

Markedly different cytotoxic and cytogenetic activities of water-soluble Cr(VI), as potassium dichromate, and Cr(III), as chromium chloride, were shown in our laboratory, with mammalian cell cultures, on the basis of their effects on the physicochemical properties of nucleic acids (Tamino & Peretta, 1980; Tamino *et al.*, 1981), nucleoside uptake and nucleic acid synthesis (Levis *et al.*, 1978*a*, *b*; Bianchi *et al.*, 1979, 1980), the functions of plasma-membrane enzymes (Luciani *et al.*, 1979), the cell-division cycle (Majone & Rensi, 1979), the induction of chromosome aberrations and sister-chromatid exchanges (Majone & Levis, 1979). A screening of the cytotoxic and clastogenic action of 11 water-soluble compounds of Cr(VI) and Cr(III) has been also carried out (Levis & Majone, 1979) and is extended here by another 10 very soluble, barely soluble and highly insoluble compounds containing Cr(VI) and Cr(III), among which are several industrial pigments the carcinogenicity of which in the rat (Maltoni, 1976) and mutagenicity in bacteria (Petrilli & De Flora, 1978*a*, *b*; De Flora, 1981) have already been established (Table I).

### MATERIALS AND METHODS

*Cells.*—The heteroploid BHK 21 Syrian hamster fibroblast line adapted to growth *in vitro* in both monolayer and suspension, the heteroploid HEp-2 human epithelial-like line and the pseudodiploid CHO Chinese hamster fibroblast line, maintained *in vitro* as monolayers, were grown in Eagle's minimal essential medium (MEM) supplemented with 10% calf serum and routinely used as already described (Levis *et al.*, 1978*a*,*b*; Levis & Majone, 1979).

*Cell growth and labelling.*—The reduction in cell growth, based on the nucleic acid content (DNA+RNA) of treated cultures, and the inhibition of DNA, RNA and protein synthesis, based on the incorporation of labelled precursors, were determined, to evaluate the cytotoxic effects of Cr compounds in BHK cell cultures. Labelling with 2 μCi/ml thymidine-6-H3 (Radiochemical Centre, Amersham, England; 2 Ci/mM), uridine-5-H3 (2–5 Ci/mM) and L-leucine-4,5-H3 (0·5–1 Ci/mM), differential extraction and determination of soluble nucleotides and amino acids, nucleic acids and proteins, and evaluation of the actual rates of macromolecular syntheses and changed uptake of soluble precursor due to Cr action, were performed by the procedures already described (Levis *et al.*, 1978*a*,*b*; Levis & Majone, 1979).

*Mitotic studies.*—HEp cell cultures were grown on coverslips, fixed in Carnoy's fluid and stained with Mayer's haemalum, as already described (Majone & Rensi, 1979). Three to four thousand cells were then examined for each dose level and each time interval, and the mitotic index (the ratio of all mitoses to the total number of cells) and the frequencies of each mitotic phase (the ratios of cells in each phase to total mitoses) were determined.

*Chromosome preparations.*—$3 \times 10^{-5}$M bromodeoxyuridine (Sigma, St Louis, Mo., U.S.A.) was incorporated for 2 cell cycles (32 h) in CHO cell cultures, and then metaphase cells were prepared as detailed elsewhere (Levis & Majone, 1979), so that chromosome aberrations and sister-chromatid exchanges (SCE) were scored on the same cell preparations.

*Chromium determinations.*—Cr(VI) was determined spectrophotometrically by the direct coloured reaction with 1,5-diphenylcarbazide (DPCA) (Riedel De Haën, Hannover, West Germany), and Cr(III) by the same reaction after oxidation to Cr(VI) with potassium permanganate, as already described (Levis *et al.*, 1978*a*, *b*; Levis & Majone, 1979). The colorimetric method was sensitive to 0·01 μg Cr(VI) per ml final solution with 5cm spectrophotometric cells, and followed Beer's law up to a concentration of 1 μg Cr(VI) per ml final solution (or 1 pt/$10^6$). Standard calibration curves, made with potassium dichromate and chromium chloride as highly soluble Cr(VI) and Cr(III) salts, gave reliable results for the direct Cr(VI) determination by DPCA in Hanks' balanced salt solution

(HBSS) and MEM, provided that phenol red was not added to the solutions as an indicator of pH, as well as for total Cr determination (Cr(VI)+Cr(III)) in HBSS, MEM and treated cells, after wet decomposition of the samples by mineralization at 180°C for 2–3 h with nitric acid: sulphuric acid: perchloric acid (3:1:1) and oxidation with potassium permanganate. The last procedure has been used also for Cr(VI) and Cr(III) determination in biological samples both by atomic absorption spectrophotometry (Feldman *et al.*, 1967) and gas chromatography (Savory *et al.*, 1970) but in our experience it does not allow us to estimate the original oxidation state of Cr in the material examined. The high-temperature acidic digestion produces significant losses of Cr(VI) and an almost complete oxidation of Cr(III) that cannot be avoided by changing the composition of the acidic mixture. Reliable estimations of Cr(VI) and Cr(III) in the treated cells have been obtained in the present investigation by suspending the cells in water after treatment, and precipitating them by 5% (final) trichloroacetic acid. According to the standard calibration curves carried out in this way, Cr(VI) can be determined directly by DPCA in the supernatant, whereas Cr(III) can be measured after hot acidic digestion and oxidation with potassium permanganate of the whole sample.

*Chromium compounds and cell treatments.*—The tested compounds of Cr(VI) and Cr(III) and their physico-chemical properties are listed in Table I. In many experiments in which most of them were highly insoluble or only partially soluble (see Tables I, IV, V), Cr compounds were directly suspended in the growth medium used for treatments. In the experiments on the induction of chromosome aberrations and SCE in CHO cells, almost complete solubilization was obtained in N HCl for Cr(III) compounds and 0·5N NaOH for Cr(VI) compounds, after which they were diluted with MEM to the final concentrations specified in Table IX. Several treatments were performed by pre-incubating the dif-

TABLE I.—*Physico-chemical properties of chromium compounds*

| Compound* | Main Cr component | Oxidation state of Cr | Chemical composition (%)† | Solubility in water |
|---|---|---|---|---|
| Chromium yellow | Lead chromate | (VI) | $PbCrO_4$ (41–85); $PbSO_4$ (4–45) $SiO_2$ (0·1–3); $Al_2O_3$ (2–6) | Very low |
| Chromium orange | Basic lead chromate | (VI) | $PbCrO_4.PbO$ | Very low |
| Molybdenum orange | Lead chromate | (VI) | $PbCrO_4$ (72–77); $PbSO_4$ (4–6) $PbMoO_4$ (12–14); $Al_2O_3$ (2) | Very low |
| Zinc yellow | Basic zinc chromate | (VI) | $ZnCrO_4.Zn(OH)_2$ (90); $CrO_3$ (10) | Slight |
| Potassium dichromate | — | (VI) | $K_2Cr_2O_7$ | High‡ |
| Neochromium | Basic chromium sulphate | (III) | $Cr(OH)SO_4$ (56–58); $Na_2SO_4$ (23–24); $H_2O$ (18–21) | Slight |
| Chromium alum | Chromium sulphate | (III) | $Cr_2(SO_4)_3$ (37–39); $K_2SO_4$ (16–18); $H_2O$ (43–47) | Slight |
| Chromite | Chromium oxide | (III) | $Cr_2O_3$ (44–46); $Fe_2O_3$ (29–30); $Al_2O_3$ (15–16); $SiO_2$ (0·5–3); CaO (0·5–2) | Very low |
| Chromium sulphate | — | (III) | $Cr_2(SO_4)_3.nH_2O$ | Very low |
| Chromium chloride | — | (III) | $CrCl_3.6H_2O$ | High‡ |

* Potassium dichromate (Mallinckrodt, St Louis, Mo, U.S.A.), chromium sulphate (Mallinckrodt) and chromium chloride (Merck, Darmstadt, West Germany) were analytical-grade reagents. Other compounds were industrial pigments, kindly supplied by Dr Cesare Maltoni (Istituto di Oncologia "Felice Addarii", Bologna, Italy), which had already been tested for carcinogenicity in the rat (Maltoni, 1976) and mutagenicity in bacteria (Petrilli & de Flora, 1978*a*,*b*; de Flora, 1981).

† As reported by the producers (Montedison, Allessandria, Italy).

‡ Up to $10^{-1}$M in water and up to $10^{-3}$M in growth medium.

ferent Cr compounds in MEM, with or without BHK cell suspensions; then the cells were discarded and the medium filtered through Millipore filters (0·22 μm pore size) and used to treat BHK or HEp cell monolayers (see Tables IV, V, VII and Fig. 3). The cytotoxic and clastogenic effects were usually determined just at the end of Cr treatments, but sometimes the treated cells were washed with HBSS and incubated with normal growth medium, so that the effects of Cr were determined at different times after the end of treatment (see Fig. 3 and Table VII). Owing to the approximate chemical composition and the very different water solubilities of the compounds used, treatment concentrations are generally expressed as mg/ml of the whole compound, whereas the concentrations of Cr(VI) or Cr(III) are specified when solubilized Cr amounts were determined by the DPCA reaction.

### RESULTS

### *Cytotoxicity of chromium on BHK cells*

Typical patterns of inhibition of macromolecular synthesis in BHK monolayers treated for different times with different concentrations of soluble Cr(VI) as potassium dichromate, are shown in Fig. 1, and in Table II the results of several such kinetic studies have been extrapolated in order to calculate the length of treatment giving equal inhibitions of DNA, RNA and protein syntheses. As already observed (Levis *et al.*, 1978*b*) Cr(VI) differentially inhibits macromolecular syntheses, producing a very rapid blockage of DNA replication, whereas RNA synthesis is reduced more slowly and only secondarily, and protein synthesis is affected even later and less. The cytotoxicity is more marked when the cells are treated in HBSS, in which Cr(VI) reduction by the extracellular components is minimal.

The action of Cr(VI) and Cr(III) compounds on growth and macromolecular syntheses of BHK cells is shown in Table III. In this experiment different concentrations of Cr compounds were added directly to the growth medium of cell suspensions, and their effects were determined 48 h later. The concentrations used were 0·5, 0·15 and 0·05 mg/ml of Cr(VI) compounds and only 0·5 mg/ml for Cr(III) compounds, on account of the already observed much lower cytotoxicity of Cr(III) (Levis *et al.*, 1978*a*; Levis & Majone, 1979). Exposure to all the concentrations of potassium dichromate and zinc yellow, as well as to 0·5 mg/ml of Cr orange, produces the maximum cytotoxicity of Cr(VI) treatments, all the cells being killed, whereas the same effect is produced only by exposure to 0·5 mg/ml of chromite, of the Cr(III) compounds (Table III). Treatments with 0·15 mg/ml of Cr yellow and molybdenum orange significantly inhibit cell growth and macromolecular syntheses, whereas neochromium, Cr alum and Cr sulphate produce comparable inhibition at a higher concentration (0·5 mg/ml). On the other hand Cr chloride, though the only completely soluble Cr(III) compound, has no cytotoxicity. DNA synthesis is the most sensitive to all Cr compounds, RNA and protein syntheses being always less inhibited.

As most of the compounds were scarcely soluble or even highly insoluble in water, preincubation for 48 h in MEM with BHK cell suspensions or in MEM without cells was carried out, to facilitate solubilization of Cr(VI) and complex formation between Cr(III) and medium components or cell metabolites. At the end of preincubation, the cells were discarded and the solubilized Cr(VI) and Cr(III) contents were determined in the filtered MEM, which was then used to treat fresh BHK monolayers for 2 h. Table IV shows the effects of preincubated Cr compounds on macromolecular syntheses, and the amounts of Cr(VI) and Cr(III) solubilized in MEM in the treatments. Among Cr(VI) compounds, potassium dichromate and zinc yellow again display the highest cytotoxicity, as they markedly inhibit DNA synthesis even when preincubated at 0·05 mg/ml. Moreover their inhibitory effects are more marked when preincubation is made in cell-free MEM. Among Cr(III) compounds, chromite is the most powerful, as it sig-

FIG. 1.—Effects of Cr(VI), as potassium dichromate, on macromolecular syntheses in BHK cell cultures. BHK monolayers were treated for different times with 1 μg/ml (□), 10 μg/ml (○, ●) and 100 μg/ml (▲) in MEM (closed symbols) or HBSS (open symbols). The actual rates of DNA (A), RNA (B) and protein (C) syntheses were determined by labelling for 1 h with tritiated precursors just at the end of treatment.

TABLE II.—*Effects of Cr(VI), as potassium dichromate, on macromolecular syntheses in BHK monolayers*

| $K_2Cr_2O_7$ treatment for various times | Macro-molecular synthesis* | Length of treatment (h) giving comparable inhibition† 50% | > 90% |
|---|---|---|---|
| 100 μg/ml in MEM | DNA | < 0·25 | < 0·75 |
| | RNA | 0·75–1·0 | 5–7 |
| | Proteins | 1–3 | 6–10 |
| 10 μg/ml in HBSS | DNA | 0·25–0·5 | 2–3 |
| | RNA | 1–2 | 6–7 |
| | Proteins | 3–5 | 8–10 |
| 10 μg/ml in MEM | DNA | 0·5–1·0 | 2–3 |
| | RNA | 2–4 | > 10 |
| | Proteins | 5–9 | > 10 |

* The actual rates of syntheses were calculated by labelling for 1 h with tritiated precursors just at the end of treatment.

† Calculated by extrapolation of macromolecular synthesis-inhibition kinetics (Fig. 1). The range in 4 separate experiments is given.

nificantly inhibits DNA and RNA, but not protein synthesis. The other Cr(VI) and Cr(III) compounds, however, are scarcely active even at 0·5 mg/ml, as they partially inhibit nucleic acid synthesis only when preincubated in cell-free MEM, with the exception of $CrCl_3$ which is completely inactive throughout.

Concerning the amounts of Cr solubilized during preincubation, it can be observed (Table IV) that potassium dichromate and $CrCl_3$ are completely soluble in MEM; 0·5 mg/ml of these compounds correspond to 176·9 μg/ml of Cr(VI) and 97·7 μg/ml of Cr(III) respectively, which are very close to the observed values. Partial solubilization of Cr(III) is obtained with neochromium and Cr alum, whereas preincubation of the other compounds gives very low levels of soluble Cr. Among

TABLE III.—*Cytotoxic effects of Cr compounds in BHK monolayers*

| Treatment (48 h in MEM) | Concentration (mg/ml) | Cell growth (% of controls)* | Macromolecular syntheses (% of controls)† DNA | RNA | Proteins |
|---|---|---|---|---|---|
| Chromium yellow (VI) | 0·5 | 65 | 14 | 45 | 83 |
| | 0·15 | 86 | 65 | 87 | 100 |
| | 0·05 | 100 | 86 | 90 | 100 |
| Chromium orange (VI) | 0·5 | 0 | 0 | 0 | 0 |
| | 0·15 | 41 | 11 | 53 | 75 |
| | 0·05 | 72 | 43 | 84 | 92 |
| Molybdenum orange (VI) | 0·5 | 48 | 15 | 34 | 56 |
| | 0·15 | 72 | 52 | 65 | 100 |
| | 0·05 | 92 | 75 | 76 | 100 |
| Zinc yellow (VI) | 0·5/0·15/0·05 | 0 | 0 | 0 | 0 |
| Potassium dichromate (VI) | 0·5/0·15/0·05 | 0 | 0 | 0 | 0 |
| Neochromium (III) | 0·5 | 75 | 48 | 69 | 88 |
| Chromium alum (III) | 0·5 | 88 | 66 | 89 | 96 |
| Chromite (III) | 0·5 | 0 | 0 | 0 | 0 |
| Chromium sulphate (III) | 0·5 | 68 | 45 | 55 | 69 |
| Chromium chloride (III) | 0·5 | 100 | 100 | 100 | 100 |

* Estimated as DNA + RNA content of cultures.
† Estimated by incubation for 1 h with labelled precursors just at the end of treatment.

TABLE IV.—*Cytotoxic effects of Cr compounds in BHK monolayers (cont.)*

| Treatment* | Concentration during preincubation (mg/ml) | Macromolecular syntheses (% of controls)† Preinc. in MEM DNA | RNA | Proteins | Preinc. in MEM + BHK DNA | RNA | Proteins | Chromium solubilized (μg/ml) at end of preincubation In MEM Cr (VI) | Cr (III) | In MEM + BHK Cr (VI) | Cr (III) |
|---|---|---|---|---|---|---|---|---|---|---|---|
| Chromium yellow (VI) | 0·5 | 40 | 59 | 72 | 100 | 100 | 100 | 3·8 | 0·8 | 0·2 | 3·1 |
| | 0·15/0·05 | 100 | 100 | 100 | 100 | 100 | 100 | — | — | — | — |
| Chromium orange (VI) | 0·5 | 62 | 93 | 100 | 100 | 100 | 100 | 2·7 | 0·4 | 0·0 | 3·5 |
| | 0·15/0·05 | 100 | 100 | 100 | 100 | 100 | 100 | — | — | — | — |
| Molybdenum orange (VI) | 0·5 | 74 | 81 | 100 | 100 | 100 | 100 | 3·4 | 1·7 | 0·3 | 4·7 |
| | 0·15/0·05 | 100 | 100 | 100 | 100 | 100 | 100 | — | — | — | — |
| Zinc yellow (VI) | 0·5 | 3 | 17 | 24 | 4 | 25 | 40 | 6·9 | 1·1 | 2·8 | 15·8 |
| | 0·15 | 3 | 26 | 47 | 4 | 42 | 62 | — | — | — | — |
| | 0·05 | 36 | 94 | 100 | 52 | 100 | 100 | — | — | — | — |
| Potassium dichromate (VI) | 0·5 | 4 | 39 | 52 | 7 | 49 | 69 | 166·2 | 17·1 | 134·0 | 45·5 |
| | 0·15 | 4 | 42 | 68 | 16 | 64 | 100 | — | — | — | — |
| | 0·05 | 24 | 90 | 100 | 35 | 100 | 100 | — | — | — | — |
| Neochromium (III) | 0·5 | 85 | 93 | 100 | 100 | 100 | 100 | 0·0 | 35·2 | 0·0 | 48·0 |
| Chromium alum (III) | 0·5 | 92 | 100 | 100 | 100 | 100 | 100 | 0·0 | 38·3 | 0·0 | 48·5 |
| Chromite (III) | 0·5 | 53 | 84 | 100 | 68 | 96 | 100 | 5·3 | 1·1 | 1·6 | 11·1 |
| Chromium sulphate (III) | 0·5 | 82 | 87 | 100 | 100 | 100 | 100 | 0·0 | 2·8 | 0·0 | 3·1 |
| Chromium chloride (III) | 0·5 | 100 | 100 | 100 | 100 | 100 | 100 | 0·0 | 111·9 | 0·0 | 114·3 |

* Cells exposed for 24 h to growth medium preincubated for 48 h with different concentrations of Cr compounds.
† Estimated as in Table III.

TABLE V.—*Cytotoxic effects of Cr(VI), as zinc yellow and chromium orange, in BHK monolayers*

| | | Macromolecular syntheses (% of controls) | | | | | | Chromium solubilized (μg/ml) at end of preincubation | | | |
|---|---|---|---|---|---|---|---|---|---|---|---|
| | | Preinc. in MEM | | | Preinc. in MEM+BHK | | | In MEM | | In MEM+BHK | |
| Treatment* (0·5 mg/ml) | Preincubation | DNA | RNA | Proteins | DNA | RNA | Proteins | Cr (VI) | Cr (III) | Cr (VI) | Cr (III) |
| Zinc yellow (VI) | 2 h | 10 | 54 | 79 | 21 | 64 | 100 | — | — | — | — |
| | 1 d | 8 | 40 | 62 | 16 | 49 | 94 | 6·1 | 0·6 | 0·2 | 9·3 |
| | 2 d | 4 | 19 | 37 | 6 | 28 | 62 | 4·8 | 0·3 | 1·7 | 8·8 |
| | 3 d | 4 | 12 | 32 | 5 | 20 | 56 | 6·2 | 0·5 | 1·7 | 12·2 |
| | 6 d | 1 | 9 | 25 | 2 | 17 | 41 | 6·9 | 0·9 | 1·8 | 15·8 |
| Chromium orange (VI) | 2 h | 100 | 100 | 100 | 100 | 100 | 100 | — | — | — | — |
| | 1 d | 84 | 100 | 100 | 95 | 100 | 100 | 0·5 | 0·2 | 0·0 | 2·1 |
| | 2 d | 54 | 85 | 92 | 86 | 96 | 100 | 0·9 | 0·4 | 0·0 | 2·8 |
| | 3 d | 47 | 81 | 100 | 78 | 100 | 100 | 0·9 | 0·6 | 0·2 | 3·2 |
| | 2 d | 21 | 66 | 78 | 52 | 72 | 92 | 1·1 | 0·5 | 0·2 | 3·9 |

* Monolayers were exposed for 24 h to preincubated growth medium.

the water-insoluble Cr compounds, zinc yellow and chromite give the highest solubilized chromium levels, particularly when preincubated with BHK cells. As a general rule, preincubation of Cr(VI) compounds in cell-free MEM solubilizes greater amounts of Cr(VI), whereas preincubation in MEM with cells is accompanied by almost complete reduction of solubilized Cr(VI) to Cr(III). It must be stressed that, among Cr(III) compounds, only preincubation of chromite gives, on solubilization, significant amounts of Cr(VI), obviously derived from impurities in the chromite.

The cytotoxic effects of the 2 poorly soluble Cr(VI) compounds, zinc yellow and Cr orange, have been determined in BHK monolayers after different durations of preincubation in MEM, with or without cell suspensions (Table V). Zinc yellow strongly inhibits nucleic acid synthesis even after 2h preincubation, and its cytotoxicity increases with longer preincubations. Cr orange is much less active, giving considerable inhibition of nucleic acid synthesis only after 6 days of preincubation. Again the cytotoxicity is greater after preincubation in cell-free MEM than in MEM with cells. An increasing amount of Cr(VI) is solubilized with increasing preincubation time in MEM, and is reduced to Cr(III) in the presence of cells. It appears also in Table V that preincubation in MEM with cells gives more solubilized total Cr than preincubation in MEM alone, which is an almost general rule for all Cr compounds used (see Table IV).

*Chromium accumulation and stability in BHK cells*

The accumulation of Cr in BHK cell suspensions was determined after exposure for up to 6 h to the very soluble Cr(VI) and Cr(III) compounds potassium dichromate and $CrCl_3$ (Fig. 2). Even after 6h incubation with 100 μg/ml of Cr(VI), significant amounts of oxidized Cr cannot be detected by direct reaction with DPCA in the treated cells. On the contrary, increasing amounts of Cr(III) are accumulated in the cells with increasing incubation times, and with increasing treatment concentrations of Cr(VI) or Cr(III). The amounts of cell-linked Cr are much higher when treatment with Cr(VI) is made in MEM than in HBSS, whereas just the opposite is true for treatments with Cr(III). Moreover, much more Cr is accumulated in cells incubated with Cr(III) than with the same concentrations of Cr(VI).

A considerable fraction of the Cr

FIG. 2.—Accumulation of Cr(III) in BHK cell cultures. Cr(III) amounts in cell suspensions were determined after different lengths of treatment with 1 µg/ml in HBSS (□), 10 µg/ml in HBSS( ○) or MEM (●) and 100µg/ml in HBSS (△) or MEM (▲) of Cr(VI) as potassium dichromate (A), or Cr(III) as $CrCl_3$ (B). Chromium amounts are expressed as µg of Cr(III) per $10^2$ µg of DNA, corresponding to $\sim 10^7$ BHK cells suspended in 20 ml of medium.

TABLE VI.—*Stability of Cr(III) accumulated in BHK cells*

| | | Incubation time (h)† | | | | |
|---|---|---|---|---|---|---|
| Treatment* | Fraction | 0 | 12 | 24 | 36 | 48 |
| Potassium dichromate (VI) (100 µg/ml) | Growth medium | — | 1·5 | 1·7 | 1·3 | 2·0 |
| | Cells | 3·1 | 1·4 | 1·6 | 1·6 | 1·5 |
| | Total | 3·1 | 2·9 | 3·3 | 2·9 | 3·5 |
| Chromium chloride (III) (10 µg/ml) | Growth medium | — | 7·0 | 8·8 | 6·0 | 9·4 |
| | Cells | 33·3 | 26·1 | 19·0 | 29·2 | 22·5 |
| | Total | 33·3 | 33·1 | 27·8 | 35·2 | 35·2 |

* Cell suspensions were treated for 6 h in MEM, then washed with HBSS and incubated in normal growth medium.

† Chromium levels are expressed as µg of Cr(III) per 10 µg of DNA (corresponding to $\sim 10^7$ BHK cells, suspended in 20 ml of growth medium).

accumulated in BHK cells during a 6h treatment with Cr(VI) or Cr(III) remains firmly bound to the cells, even when they are washed and re-incubated for up to 48h in normal growth medium (Table VI). This fraction is about 50 or 75% of the Cr accumulated in the cells during treatment with Cr(VI) or Cr(III), respectively.

*Cytotoxicity of chromium in HEp cells*

The cytotoxicity of Cr on mitosis of HEp cells has been determined by treatment with MEM preincubated for different times with Cr(VI) and Cr(III) compounds. Fig. 3 shows the effects of zinc yellow: the percentage of total mitoses decreases after treatment, the more markedly and rapidly the longer the preincubation, indicating that mitotic inhibition is produced by the solubilized Cr. On the other hand the frequencies of abnormal prophases and metaphases are *increased* just

FIG. 3.—Effects of Cr(VI), as zinc yellow, on mitosis of HEp cells. The frequencies of total mitoses (A), and abnormal prophases (B) and metaphases (C) have been determined at different intervals of time after HEp monolayers had been exposed for 2 h to growth medium preincubated for 1 (●), 2 (○), 3 (▲) or 6 (△) days with 0·5 mg/ml of zinc yellow, in the presence of BHK cell suspensions.

after treatment and fall later, showing that, besides late mitotic inhibition, blockage of the cell cycle during mitosis is also produced. As summarized in Table VII, the differential cytotoxicity of the tested Cr compounds on growth and macromolecular syntheses of BHK cells (see above) very closely parallels their effect on HEp cells.

*Clastogenic effects of chromium in CHO cells*

The frequencies of chromosome aberrations and SCE have been determined in CHO cells after incubation for 2 division cycles with different concentrations of Cr compounds added directly to the growth medium (Table VIII). The mean number of chromosome aberrations is significantly increased after treatment with all Cr(VI) compounds, being 2–3 times the control values after exposure to 5–25 $\mu$g/ml of the insoluble or poorly soluble Cr(VI) pigments and 0·1 $\mu$g/ml water-soluble Cr(VI) as potassium dichromate (0·3 $\mu$g/ml of salt)—an increase of the same order as that found with the same concentration of other soluble Cr(VI) salts (Levis & Majone, 1979). Treatment with 150 $\mu$g/ml Cr(VI) pigments did not permit the analysis of chromosome aberrations, because of the marked inhibition of cell growth and the severe delay of the mitotic cycle. The frequency of chromosome aberrations is also increased after treatment with Cr(III) compounds, being about doubled after exposure to 25–150 $\mu$g/ml. On the other hand, the frequencies of SCE are significantly increased by all Cr(VI) compounds and by chromite, but not by the other Cr(III) compounds. The increase is of the same order as that already obtained with other Cr(VI) salts, and it is much lower than that induced by mitomycin C, which was the positive control for the response of our cell system to the induction of chromosome damage (Levis & Majone, 1979).

In a parallel experiment, CHO cells have been exposed to Cr(VI) and Cr(III) compounds previously solubilized with HCl and NaOH and then diluted in the medium used for treatments. It can be seen (Table IX) that Cr(VI) compounds induce chromosome aberrations at the same concentration of water-soluble Cr(VI) (*i.e.* 0·1–0·3 $\mu$g/ml) provided that they are solubilized with NaOH. Cr(III)

TABLE VII.—*Effects of Cr compounds on mitosis of HEp cells*

| Treatment* | Mitotic inhibition: Early† | Mitotic inhibition: Late‡ | Mitotic block§ | Caryological alterations‖ |
|---|---|---|---|---|
| Chromium yellow (VI) | ++ | +++ | ++ | + |
| Chromium orange (VI) | ++ | +++ | ++ | + |
| Molybdenum orange (VI) | ++ | +++ | ++ | + |
| Zinc yellow (VI) | +++ | +++ | +++ | + |
| Potassium dichromate (VI) | +++ | +++ | +++ | + |
| Neochromium (III) | − | + | − | + |
| Chromium alum (III) | + | + | + | + |
| Chromite (III) | ++ | +++ | ++ | + |
| Chromium sulphate (III) | + | + | ++ | + |
| Chromium chloride (III) | + | + | + | + |

* Cell monolayers were exposed for 2 h to growth medium preincubated for 2 days with 0·5 mg/ml of Cr compounds in the presence of BHK cell suspensions.

† % Reduction of normal prophases at the end of treatment: +++ (>70%); ++ (30–70%); + (<30%); − (nil).

‡ % Reduction of normal prophases 24 h after treatment.

§ % Increase of abnormal metaphases 2–4 h after treatment: +++ (>100%); ++ (50–100%); + (<50%); − (nil).

‖ Mainly chromosome bridges at anaphase; micronuclei and multinucleated cells at interphase; sticky, lagging and pulverized chromosomes at metaphase. Details as in Tables VII–VIII.

TABLE VIII.—*Chromosome aberrations and sister chromatid exchanges induced by Cr(VI) and Cr(III) compounds in CHO cell cultures*

| Treatment* | Concentrations (μg/ml) | Cell growth† (% control) | Metaphases counted‡ | Chromosome and chromatid aberrations per 100 metaphases | SCE/metaphase | *t* for SCE/metaphase§ | *P* |
|---|---|---|---|---|---|---|---|
| — | — | 100 | 61 | 13·3 | 7·47±0·11 | — | — |
| Chromium yellow (VI) | 5 | 85 | 95 | 29·2 | 9·34±0·19 | 7·42 | <0·001 |
| | 25 | 78 | 40 | 22·5 | 9·90±0·29 | 9·11 | <0·001 |
| | 150 | 48 | — | — | — | — | — |
| Chromium orange (VI) | 5 | 100 | 75 | 25·0 | 9·36±0·25 | 6·50 | <0·001 |
| | 25 | 93 | 30 | 26·7 | 10·47±0·29 | 11·66 | <0·001 |
| | 150 | 56 | — | — | — | — | — |
| Molybdenum orange (VI) | 5 | 100 | 50 | 20·0 | 10·48±0·38 | 8·33 | <0·001 |
| | 25 | 100 | 45 | 24·4 | 10·11±0·36 | 8·01 | <0·001 |
| | 150 | 85 | 25 | 26·0 | 9·76±0·13 | 12·28 | <0·001 |
| Zinc yellow (VI) | 5 | 73 | 14 | 28·6 | 13·14±1·10 | 10·11 | <0·001 |
| | 25 | 29 | — | — | — | — | — |
| | 150 | 10 | — | — | — | — | — |
| Potassium dichromate (VI) | 0·3 | 83 | 64 | 32·5 | 12·84±0·51 | 10·08 | <0·001 |
| Neochromium (III) | 5 | 100 | 30 | 20·0 | 7·46±0·22 | 0·05 | >0·7 |
| | 25 | 100 | 30 | 23·3 | 7·57±0·18 | 0·50 | >0·5 |
| | 150 | 100 | 30 | 26·7 | 7·62±0·22 | 0·69 | >0·4 |
| Chromium alum (III) | 5 | 100 | 40 | 15·0 | 7·21±0·18 | 1·25 | >0·2 |
| | 25 | 100 | 40 | 20·0 | 7·40±0·20 | 0·34 | >0·7 |
| | 150 | 100 | 28 | 28·6 | 7·53±0·31 | 0·23 | >0·7 |
| Chromite (III) | 5 | 100 | 65 | 26·5 | 9·49±0·31 | 6·02 | <0·001 |
| | 25 | 100 | 62 | 23·4 | 8·27±0·30 | 2·52 | <0·02 |
| | 150 | 88 | — | — | — | — | — |
| Chromium sulphate (III) | 5 | 100 | 40 | 10·0 | 7·10±0·17 | 1·80 | >0·05 |
| | 25 | 100 | 40 | 22·5 | 7·45±0·27 | 0·79 | >0·4 |
| | 150 | 93 | 60 | 25·0 | 7·73±0·35 | 0·72 | >0·4 |
| Chromium chloride (III) | 50 | 95 | 34 | 29·4 | 7·20±0·41 | 0·81 | >0·4 |

* Monolayers were treated for 32 h in MEM.

† Cell growth was estimated on the basis of the DNA+RNA content of treated cultures.

‡ Chromosome aberrations and SCE were scored on the same 2nd division metaphases.

§ *t* values for comparison with control.

TABLE IX.—*Chromosome aberrations and sister chromatid exchanges induced by Cr(VI) and Cr(III) compounds in CHO cell cultures*

| Treatment* | Concentration (μg/ml) | Metaphases counted† | Chromosome and chromatid aberrations per 100 metaphases | SCE/metaphase | t for SCE/metaphase‡ | P |
|---|---|---|---|---|---|---|
| — | — | 50 | 10·0 | 7·54 ± 0·16 | — | — |
| HCl | 0·010N | 40 | 10·0 | 7·43 ± 0·18 | — | — |
| | 0·025N | 45 | 10·0 | 7·71 ± 0·19 | — | — |
| NaOH | 0·005N | 30 | 10·0 | 6·87 ± 0·13 | — | — |
| | 0·015N | 40 | 12·5 | 7·81 ± 0·23 | — | — |
| Chromium yellow (VI) | 0·1 | 15 | 26·6 | 8·53 ± 0·24 | 6·50 | < 0·001 |
| | 0·3 | 20 | 20·0 | 8·85 ± 0·28 | 2·70 | < 0·01 |
| Chromium orange (VI) | 0·1 | 40 | 15·0 | 9·12 ± 0·21 | 6·92 | < 0·001 |
| | 0·3 | 40 | 15·0 | 9·35 ± 0·24 | 3·39 | < 0·01 |
| Molybdenum orange (VI) | 0·1 | 40 | 15·0 | 9·12 ± 0·22 | 8·20 | < 0·001 |
| | 0·3 | 38 | 15·0 | 9·89 ± 0·27 | 5·87 | < 0·001 |
| Zinc yellow (VI) | 0·1 | 40 | 12·5 | 9·25 ± 0·28 | 6·85 | < 0·001 |
| | 0·3 | 30 | 19·9 | 9·17 ± 0·36 | 3·39 | < 0·01 |
| Potassium dichromate (VI) | 0·1 | 46 | 15·2 | 10·26 ± 0·35 | 7·60 | < 0·001 |
| | 0·3 | 30 | 23·3 | 11·10 ± 0·60 | 5·60 | < 0·001 |
| Neochromium (III) | 10 | 40 | 12·5 | 7·37 ± 0·16 | 0·25 | > 0·7 |
| | 25 | 40 | 10·0 | 7·42 ± 0·16 | 1·13 | > 0·2 |
| Chromium alum (III) | 10 | 47 | 7·5 | 7·13 ± 0·14 | 1·33 | > 0·1 |
| | 25 | 40 | 12·5 | 7·24 ± 0·17 | 1·72 | > 0·1 |
| Chromite (III) | 10 | 33 | 17·5 | 8·81 ± 0·22 | 4·80 | < 0·001 |
| | 25 | 40 | 15·0 | 9·60 ± 0·21 | 6·64 | < 0·001 |
| Chromium sulphate (III) | 10 | 40 | 10·0 | 6·68 ± 0·16 | 1·15 | > 0·2 |
| | 25 | 40 | 10·0 | 7·30 ± 0·15 | 1·64 | > 0·2 |
| Chromium chloride (III) | 10 | 50 | 12·5 | 6·86 ± 0·28 | 1·62 | > 0·1 |
| | 25 | 40 | 10·0 | 7·52 ± 0·26 | 0·65 | > 0·5 |

* Monolayers were treated for 32 h with Cr compounds previously dissolved in HCl (for Cr(III)) or NaOH (for Cr(IV)) and then diluted in MEM (final concentrations of HCl in MEM: 0·010–0·025; NaOH: 0·005–0·015N).

† Chromosome aberrations and sister chromatid exchanges were scored in the same 2nd division metaphases.

‡ *t* values for comparison with controls treated either with HCl (0·010N, 0·025N) or NaOH (0·005N, 0·015N).

compounds solubilized with HCl and used at the concentrations of 10–25 μg/ml are inactive, with the exception of chromite, which significantly increases the frequency of chromosome aberrations. It is confirmed that the frequency of SCE is increased by the exposure to all Cr(VI) compounds and chromite, but not to the other Cr(III) compounds.

### DISCUSSION

According to the International Agency for Research on Cancer (1980) there is "sufficient" evidence for the carcinogenicity of several Cr(VI) compounds in the rat, whereas data from experiments with Cr(III) compounds are considered still inadequate for the evaluation of their carcinogenicity. Moreover "sufficient" evidence for a high risk of lung cancer in workers engaged in the production of chromates (Cr(VI)) is also recognized by the IARC, though the true carcinogenic agents cannot be identified, as the epidemiological data do not allow the evaluation of the relative contribution to the carcinogenic risk of Cr(III) or Cr(VI) forms, both present in chromate production.

Concerning the mutagenic power of Cr compounds, several Cr(VI) salts have been shown to be active in different test systems *in vitro* and *in vivo*, as they are capable of inducing errors of nucleotide incorporation in the course of *in vitro* DNA replication (Sirover & Loeb, 1976; Tkeshelashvili *et al.*, 1980), point mutations in bacteria (Venitt & Levy, 1974; Nishioka, 1975; Green *et al.*, 1976; Petrilli & De Flora, 1978*b*; Löfroth, 1978; Nakamuro *et al.*, 1978; Nestmann *et al.*, 1979; Kada *et al.*, 1980; Kanematsu *et al.*, 1980; De Flora, 1981), yeast (Bonatti *et al.*, 1976) and mammalian cells (Newbold *et al.*, 1979); and gene conversion (Bonatti *et al.*, 1976) and mitotic recombination (Nestmann *et al.*, 1979) in yeasts; chromosomal aberrations (Tsuda & Kato, 1977; Nakamuro *et al.*, 1978; Newbold *et al.*, 1979; Umeda & Nishimura, 1979; Levis & Majone, 1979; Douglas *et al.*, 1980); SCE (Levis & Majone, 1979; Majone & Rensi, 1979; Majone & Levis, 1979; MacRae *et al.*, 1979; Douglas *et al.*, 1980); DNA damage (Whiting *et al.*, 1979; Tamino & Peretta, 1980; Douglas *et al.*, 1980; Tamino *et al.*, 1981); DNA repair synthesis (Whiting *et al.*, 1979); morphological transformation (Fradkin *et al.*, 1975; Tsuda & Kato, 1977; Di Paolo & Casto, 1979) and enhancement of viral transformation (Casto *et al.*, 1979) in cultured mammalian cells; increased frequency of micronuclei and chromosomal aberrations in cells of rodents treated *in vivo* (Wild, 1978) as well as in lymphocytes of professionally exposed workers (Bigaliev *et al.*, 1977). Cr(VI) compounds gave positive results in the mouse "spot" test (Knudsen, 1980) and transformed Syrian hamster cells when injected into pregnant hamsters (Di Paolo & Casto, 1979), thus indicating that they are also transplacental mutagens. When tested for their ability to induce cytotoxic (White *et al.*, 1979) and mutagenic effects (Petrilli & De Flora, 1978*a*; Koshi, 1979; Knudsen, 1980; Stern, 1980; De Flora, 1981), industrial pigments and welding fumes from Cr-using processes have been shown to be uniquely active, owing to the presence of Cr(VI).

On the contrary, Cr(III) salts are usually inactive in mutagenicity tests (Venitt & Levy, 1974; Nishioka, 1975; Petrilli & De Flora, 1978*b*; Kada *et al.*, 1980; Kanematsu *et al.*, 1980; De Flora, 1981) except in systems involving a direct interaction with DNA purified *in vitro*, such as damage shown by the alteration of its physico-chemical properties (Tamino & Peretta, 1980; Tamino *et al.*, 1981) and increased error frequency during replication (Sirover & Loeb, 1976; Tkeshelashvili 1980). The only frequent cytogenetic effect in mammalian cells treated *in vitro* with Cr(III) compounds is the increase of chromosome aberrations (Nakamuro *et al.*, 1978; Levis & Majone, 1979; see also the present data), but it is obtained with doses much higher than the active Cr(VI) concentrations, and could be related to an indirect effect produced by such extreme conditions, *e.g.* by the release of lysosomal nucleases. Moreover, it is found in cell lines permanently adapted to growth *in vitro*, which are characterized by chromosomal instability. Toxicity data in animals (IARC, 1980) and cytotoxicity studies in cultured mammalian cells (Levis & Majone, 1979; White *et al.*, 1979; see also the present results) accordingly show that Cr(VI) is 100 or even 1000 times more active than Cr(III).

On this basis, the cytotoxic, mutagenic and carcinogenic effects of Cr have been attributed to its oxidized state (Levis *et al.*, 1978*a*, *b*; Norseth, 1979; Bianchi *et al.*, 1979; Langard, 1980; Petrilli & De Flora, 1980; Leonard & Lauwerys, 1980). The positive mutagenicity occasionally obtained with Cr(III) compounds (Nakamuro *et al.*, 1978) cannot be related to conversion of Cr(III) to the active Cr(VI) form, which was described in plant cells (Skeffington *et al.*, 1976), but which is generally deemed not to take place in animal systems (Mertz, 1969; Norseth, 1979; Langard, 1980; Leonard & Lauwerys, 1980). The shift of Cr(III) to Cr(VI) was shown not to occur *in vitro* with different metabolic systems, and was obtained only by treating with a strong oxidizing agent such as potassium permanganate (Petrilli & De Flora, 1978*b*), so it does not appear to be reproducible by biological factors, at least in short-term *in vitro* assays. Contamination with Cr(VI) has been demonstrated for Cr(III) industrial compounds (Petrilli & De Flora, 1978*b*; see also the present data on chromite contamination) and even for reagent-grade laboratory products (Levis & Majone, 1979), which accounts for the genetic activity observed with those agents; the actual oxidation state of Cr in the compounds used in the carcinogenicity and mutagenicity tests is rarely checked. The conversion of Cr(III) to Cr(VI) in the organism has been postulated by Grogan (1957) but it should be a slow process, occurring only under particular conditions of retention and accumulation of the metal, and only in tissues with a very low reducing potential (Petrilli & De Flora, 1978*b*). This could account for the carcinogenic action exerted at the implant site by Cr(III) compounds administered i.m. or s.c. (Maltoni, 1976; IARC, 1980).

The results of the present investigation confirm the marked differences in cytotoxic and cytogenetic activity of Cr(VI) and Cr(III) which we had already noticed on treating mammalian cell cultures with water-soluble Cr compounds (Levis *et al.*, 1978*b*; Levis & Majone, 1979; Majone & Rensi, 1979; Luciani *et al.*, 1979; Bianchi *et al.*, 1980) and can contribute to a better understanding of the mechanisms of Cr carcinogenic action.

The mutagenic action of the compounds used in the present investigation has been determined in the *Salmonella*/microsome test by Petrilli & De Flora (1978*a*,*b*) and De Flora (1981), who showed that, among Cr(VI) pigments, partially soluble zinc yellow acted as a mutagen in the plate test at the same dose range as very soluble sodium dichromate and potassium chromate, and poorly soluble Cr orange and molybdenum orange were mutagens in the spot test, whereas Cr yellow displayed neither mutagenic nor toxic activity, owing to its complete insolubility in water. Cr orange, molybdenum orange and Cr yellow, as well as pure reagent-grade lead chromate, however, were positive in the plate test when dissolved in 0·5N NaOH. In the same studies, all Cr(III) compounds were inactive as mutagens except chromite, which was positive in the spot test owing to contamination with traces of soluble Cr(VI). Carcinogenicity tests have been performed with the same industrial products by Maltoni (1976), who injected them s.c. into rats. Insoluble Cr(VI) pigments, namely Cr yellow, Cr orange and molybdenum orange, raised the frequency of sarcomas at the site of injection from 0% in the controls to 65–90%, whereas Cr(III) pigments neochromium and Cr alum produced lower but significant increases (20–25%). The same chromite that was cytotoxic and clastogenic in the present study and mutagenic in the study of Petrilli & De Flora (1978*b*) owing to traces of Cr(VI), failed to induce tumours in any of the 40 animals tested by Maltoni (1976) but, as in the case of zinc yellow, necrosis was produced at the site of injection (Maltoni, personal communication), most probably due to the oxidizing action of soluble Cr(VI) in the administered compounds.

In contrast to the low cytotoxicity of Cr(III), much higher Cr levels are detected in the cells incubated with Cr(III) than with the same Cr(VI) concentrations (Fig. 2). As for treatments with Cr(VI), the amounts of cell-linked Cr are higher when the incubation is made in MEM than in HBSS, because greater amounts of Cr(III) are produced in MEM by the reduction of Cr(VI) by components of the growth medium (Levis *et al.*, 1978*b*). The opposite is found for incubations with Cr(III) (Fig. 2), probably because ligand formation with cell constituents is more limited for Cr(III) complexes involving MEM components than for Cr(III) chelates and olates present in the simplified HBSS solution (Mertz, 1969).

The different biological activities of Cr(VI) and Cr(III) are certainly related to a marked difference in Cr uptake through the cell membrane, very active mechanisms for Cr(VI) transport having been demonstrated, which are accomplished by specific chromate-ion permeases (Vallée, 1969), whereas the transport of Cr(III) complexes and chelates is extremely limited (Mertz, 1969; Norseth, 1979; Langard, 1980). A very rapid accumulation of Cr(III), even much faster than that of Cr(VI), was described in plant root cells (Skeffington *et al.*, 1976), where it was shown that most of the absorbed Cr(VI) was in the soluble fraction, while most of the Cr(III) was in the insoluble membrane fraction, as in the present experiments (see Fig. 2 and Table VI). A multiplicity of cation-binding sites in the cell walls and cell membrane could adsorb Cr(III), which may explain why it is less toxic and transported less than Cr(VI) inside the cell, though more readily accumulated on the cell surface. The present experiments (Table VI) indicate that a considerable fraction of Cr(III) (50% or 75% of Cr accumulated in the cells during treatment with Cr(VI) or Cr(III) respectively), · remains firmly bound to the cells even when they are incubated for up to 48 h in normal growth medium.

Whereas the cytotoxic action of Cr(VI) has been attributed to the oxidation of biological targets placed mainly on the cell membrane (Levis *et al.*, 1978*a,b*; Bianchi *et al.*, 1980; Luciani *et al.*, 1979), its genetic activity could depend on the interaction of intracellularly reduced Cr(III) with specific targets on the DNA molecule (Levis *et al.*, 1978*a,b*; Norseth, 1979; Langard, 1980; Petrilli & De Flora, 1980; Bianchi *et al.*, 1980; Leonard & Lauwerys, 1980). Interactions with nucleic acids, giving changes of their physicochemical and biochemical properties which could be relevant to the production of genetic changes, have been shown to occur both when purified nucleic acids are treated *in vitro* with Cr(III) compounds (Sirover & Loeb, 1976; Tamino & Peretta, 1980; Tkeshelashvili *et al.*, 1980; Tamino *et al.*, 1981) and when cells are treated with Cr(VI) compounds (Whiting *et al.*, 1979; Douglas *et al.*, 1980; Tamino *et al.*, 1981). Owing to the stability of Cr(III) chelates and coordination complexes and to their very low exchange rate with biological ligands (Mertz, 1969; Norseth, 1979; Langard, 1980; Petrilli & De Flora, 1980), the crucial site for the expression of Cr(VI) (carcino) genetic activity should be where Cr(VI) is converted to Cr(III). Only Cr(VI) reduction within the cell nucleus, and then only in the cells of target organs, could produce the interaction of Cr(III) with biological molecules relevant for the expression of carcinogenic effects. It is generally assumed that Cr exists within cells only in its reduced form, and Cr(III) is indeed detected, even after treatment with Cr(VI) compounds, by colorimetry (Levis *et al.*, 1978*a*, *b*; and in the present data), atomic absorption spectrophotometry (Feldman *et al.*, 1967) and gas chromatography (Savory *et al.*, 1970). All these procedures, however, require wet decomposition of the biological samples with acidic mixtures which, in our experience, produce significant reduction of Cr(VI) to Cr(III) (see Methods). So we cannot exclude the possibility that limited amounts of Cr(VI), not reduced within the cytoplasm, reach the cell nucleus and react there with the DNA targets. The existence of Cr(VI) within root cells treated with potassium chromate has been demonstrated by Skeffington *et al.* (1976) by high-voltage paper electrophoresis.

It is noteworthy that Cr(VI) mutagenicity is decreased and even suppressed by the rat liver microsomal fraction, erythrocyte lysates, human gastric juice (Petrilli & De Flora, 1978*a*; Löfroth, 1978; De Flora & Boido, 1980; De Flora, 1978) and through reduction to Cr(III) by simple oxidoreduction, but it is not reduced by the microsomal fraction from rat muscle (Petrilli & De Flora, 1978*a*) and it is partially reduced by the microsomal preparations from rat lung, though far less efficiently than by the corresponding liver preparations (Petrilli & De Flora, 1980). These observations correlate quite well with the preferential localization of Cr-induced cancers in the human lung, with the lack of Cr(VI) oral carcinogenicity, with the development of tumours at implant sites (*e.g.* respiratory tract and muscles) in rodents treated with Cr(VI) compounds, and with the lack of carcinogenicity of Cr(III) compounds (IARC 1980). Therefore only in the simplified *in vitro* test systems, where Cr(VI) is stable during long incubations (Bianchi *et al.*, 1979; Umeda & Nishimura, 1979) and in the cells of *in vivo* target organs such as lungs and muscles, where Cr(VI) is not reduced by the microsomal fraction, could oxidized Cr(VI) reach the cell nucleus where it could be reduced to Cr(III) on or near the critical target molecules. The very active conversion of Cr(VI) to Cr(III) by erythrocytes (Langard, 1980) and by the microsomal fraction of liver cells (De Flora, 1978; Löfroth, 1978; Petrilli & De Flora, 1978*a*) represents an important mechanism of Cr(VI) inactivation in the organism.

Only chromates of medium (Ca chromate, Zn chromate hydroxide) or low (Sr and Pb chromates) water solubility, but not the very soluble Na and K chromates and dichromates, are carcinogenic in animals (IARC, 1980) but *in vitro* chromates and dichromates of high solubility have proved to be potent cytotoxic and mutagenic agents (see above references). The obvious explanation for the lack of carcinogenicity of high-solubility chromates is that, even though they may be potential mutagens, they disappear from the site of application very rapidly, and are eventually inactivated by erythrocytes (Langard, 1980). In *in vitro* conditions we can keep compounds in the culture medium for a few days without dilution or inactivation. On the other hand lead chromate, which is known to be a fairly potent carcinogen (IARC, 1980), was shown to induce point mutations in bacteria when solubilized in acids and alkalis (Nestmann *et al.*, 1979; De Flora, 1981), but it was inactive as a mutagen for bacteria and mammalian cells in culture when used as a fine suspension in water (Newbold *et al.*, 1979; De Flora, 1981). In view of the very low water solubility of this compound and the relatively short exposure in the *in vitro* experiments, their negative result is not surprising, neither it is in conflict with the positive carcinogenicity tests where the period of exposure is much longer and the material persists at the site of application so that significant exposure of cells to chromate ions from Pb chromate would be expected.

The state of oxidation is certainly the most important parameter to be taken into account when considering the biological activity of chromium compounds, but other properties, such as the solubility in water, the ability to permeate cell membranes and the intracellular stability of the oxidized Cr(VI) form (determining different retention and diffusion rates *in vivo* and *in vitro*) are relevant in producing differential effects in long-term carcinogenicity and short-term mutagenicity tests. Although there are still definite gaps in our understanding of the relationships between chromium mutagenicity and carcinogenicity, and the exact roles of soluble and insoluble Cr(VI) and Cr(III) compounds in carcinogenesis, the knowledge of the mutagenic, cytogenetic and cytotoxic powers of Cr(VI) and Cr(III) compounds is already sufficient for assessing their respective contributions as human hazards.

This research was supported by grants from the National Research Council of Italy (Programma Finalizzato: "Promozione della Qualitá dell'-Ambiente") and the Venetia Region ("Centro Regionale di Alta Specializzazione in Cancerogenesi Ambientale").

### REFERENCES

Bianchi, V., Levis, A. G. & Saggioro, D. (1979) Differential cytotoxic activity of potassium dichromate on nucleoside uptake in BHK fibroblasts. *Chem. Biol. Interact.*, **24**, 137.

Bianchi, V., dal Toso, R., Debetto, P. & 4 others (1980) Mechanisms of chromium toxicity in mammalian cell cultures. *Toxicology*, **17**, 219.

BIGALIEV, A. B., TUREBAEV, M. N., BIGALIEVA, R. K. & ELEMESOVA, M. S. (1977) Cytogenetic examination of workers engaged in chrome production. (English abstract) *Genetika*, **13**, 545.

BONATTI, S., MEINI, M. & ABBONDANDOLO, A. (1976) Genetic effects of potassium dichromate. *Mutat. Res.*, **38**, 147.

CASTO, B. C., MEYERS, J. & DI PAOLO, J. A. (1979) Enhancement of viral transformation for the evaluation of the carcinogenic or mutagenic potential of inorganic metal salts. *Cancer Res.*, **39**, 193.

DE FLORA, S. (1978) Metabolic deactivation of mutagens in the *Salmonella*/microsome test. *Nature*, **271**, 455.

DE FLORA, S. (1981) Study of 106 organic and inorganic compounds in the *Salmonella*/microsome test. *Carcinogenesis* **2**, 283.

DE FLORA, S. & BOIDO, V. (1980) Effects of human gastric juice on the mutagenicity of chemicals. *Mutat. Res.*, **77**, 307.

DI PAOLO, J. A. & CASTO, B. C. (1979) Quantitative studies of *in vitro* morphological transformation of Syrian hamster cells by inorganic metal salts. *Cancer Res.*, **39**, 1008.

DOUGLAS, G. R., BELL, R. D., GRANT, C. E., WYTSMA, J. M. & BORA, K. C. (1980) Effect of lead chromate on chromosome aberrations, sister-chromatid exchange and DNA damage in mammalian cells *in vitro*. *Mutat. Res.*, **77**, 157.

FELDMAN, F. J., KNOBLOCK, E. C. & PURDY, W. C. (1967) The determination of chromium in biological materials by atomic absorption spectroscopy. *Anal. Chim. Acta*, **38**, 489.

FRADKIN, A., JANOFF, A., LANE, B. P. & KUSCHNER, M. (1975) *In vitro* transformation of BHK 21 cells grown in the presence of calcium chromate. *Cancer Res.*, **35**, 1058.

GREEN, M. H. L., MURIEL, W. J. & BRIDGES, B. A. (1976) Use of a simplified fluctuation test to detect low levels of mutagens. *Mutat. Res.*, **38**, 33.

GROGAN, C. H. (1957) Experimental studies in metal cancerogenesis. VIII. On the etiological factor in chromate cancer. *Cancer*, **10**, 625.

INTERNATIONAL AGENCY FOR RESEARCH ON CANCER (1980) Chromium and chromium compounds. In *Monographs on the Evaluation of the Carcinogenic Risk of Chemicals to Humans*. Vol. 23. Lyon: I.A.R.C. p. 205.

KADA, T., HIRANO, K. & SHIRASU, Y. (1980) Screening of environmental chemical mutagens by the rec-assay system with *Bacillus subtilis*. In *Chemical Mutagens: Principles and Methods for their Detection*. Vol. 6. Ed. De Serres & Hollaender. New York: Plenum Press. p. 149.

KANEMATSU, N., HARA, M. & KADA, T. (1980) Rec assay and mutagenicity studies on metal compounds. *Mutat. Res.*, **77**, 109.

KNUDSEN, I. (1980) The mammalian spot test and its use for the testing of potential carcinogenicity of welding fume particles and hexavalent chromium. *Acta Pharmacol. Toxicol.*, **47**, 66.

KOSHI, K. (1979) Effects of fume particles from stainless steel welding in sister chromatid exchanges and chromosome aberrations in cultures of Chinese hamster cells. *Indust. Health*, **17**, 39.

LANGARD, S. (1980) Chromium. In *Metals in the Environment* Ed. Waldron. New York: Academic Press. p. 111.

LEONARD, A. & LAUWERYS, R. R. (1980) Carcinogenicity and mutagenicity of chromium. *Mutat. Res.*, **76**, 227.

LEVIS, A. G., BIANCHI, V., TAMINO, G. & PEGORARO, B. (1978*a*) Cytotoxic effects of hexavalent and trivalent chromium compounds on mammalian cells *in vitro*. *Br. J. Cancer*, **37**, 386.

LEVIS, A. G., BUTTIGNOL, M., BIANCHI, V. & SPONZA, G. (1978*b*) Effects of potassium dichromate on nucleic acid and protein syntheses and on precursor uptake in BHK fibroblasts. *Cancer Res.*, **38**, 110.

LEVIS, A. G. & MAJONE, F. (1979) Cytotoxic and clastogenic effects of soluble chromium compounds on mammalian cell cultures. *Br. J. Cancer*, **40**, 523.

LÖFROTH, G. (1978) The mutagenicity of hexavalent chromium is decreased by microsomal metabolism. *Naturwissenschaften*, **65**, 207.

LUCIANI, S., DAL TOSO, R., REBELLATO, A. M. & LEVIS, A. G. (1979) Effects of chromium compounds on plasma membrane $Mg^{2+}$-ATPase activity of BHK cells. *Chem. Biol. Interact.*, **27**, 59.

LUCKEY, T. D. & VENUGOPAL, B. (1977) *Metal Toxicity in Mammals*. Vol. 2. New York: Plenum Press. p. 141.

MACRAE, W. D., WHITING, R. F. & STICH, H. F. (1979) Sister chromatid exchanges induced in cultured mammalian cells by chromate. *Chem. Biol. Interact*, **26**, 281.

MAJONE, F. & LEVIS, A. G. (1979) Chromosome aberrations and sister chromatid exchanges in Chinese hamster cells treated *in vitro* with hexavalent chromium compounds. *Mutat. Res.*, **67**, 231.

MAJONE, F. & RENSI, D. (1979) Mitotic alterations, chromosome aberrations and sister chromatid exchanges induced by hexavalent and trivalent chromium on mammalian cells *in vitro*. *Caryologia*, **32**, 379.

MALTONI, C. (1976) Predictive value of carcinogenesis bioassays. *Ann. N.Y. Acad. Sci.*, **271**, 431.

MERTZ, W. (1969) Chromium occurrence and function in biological systems. *Physiol. Rev.*, **49**, 163.

NAKAMURO, K., YOSHIKAWA, K., SAYATO, Y. & KURATA, H. (1978) Comparative studies of chromosomal aberrations and mutagenicity of trivalent and hexavalent chromium. *Mutat. Res.*, **58**, 175.

NESTMANN, E. R., MATULA, T. I., DOUGLAS, G. R., BORA, K. C. & KOWBEL, D. J. (1979) Detection of the mutagenic activity of lead chromate using a battery of microbial tests. *Mutat. Res.*, **66**, 357.

NEWBOLD, R. F., AMOS, J. & CONNELL, J. R. (1979) The cytotoxic, mutagenic and clastogenic effects of chromium-containing compounds on mammalian cells in culture. *Mutat. Res.*, **67**, 55.

NISHIOKA, H. (1975) Mutagenic activities of metal compounds in bacteria. *Mutat. Res.*, **31**, 185.

NORSETH, T. (1979) Health effects of nickel and chromium. In *Trace Metals Exposure and Health Effects*. Ed. Di Ferrante. New York: Pergamon Press. p. 135.

PETRILLI, F. L. & DE FLORA, S. (1978*a*) Metabolic deactivation of hexavalent chromium mutagenicity. *Mutat. Res.*, **54**, 139.

PETRILLI, F. L. & DE FLORA, S. (1978*b*) Oxidation of inactive trivalent chromium to the mutagenic hexavalent form. *Mutat. Res.*, **58**, 167.

PETRILLI, F. L. & DE FLORA, S. (1980) Mutagenicity of chromium compounds .In *Chromates Symposium* 80 (*ORC/IHF*). Rockville. (in press).

SAVORY, J., MUSHAK, P., SUNDERMAN, F. W., ESTES, R. H. & ROSZEL, N. O. (1970) Microdetermination of chromium in biological materials by gas chromatography. *Anal. Chem.*, **42**, 294.

SIROVER, M. A. & LOEB, L. A. (1976) Infidelity of DNA synthesis *in vitro*: Screening for potential metal mutagens or carcinogens. *Science*, **194**, 1434.

SKEFFINGTON, R. A., SHEWRY, P. R. & PETERSON, P. J. (1976) Chromium uptake in barley seedlings (*Hordeum vulgare* L.). *Planta, Berlin*, **132**, 209.

STERN, R. M. (1980) A chemical, physical and biological assay of welding fume. In *The* in Vitro *Effects of Mineral Dusts*. Ed. Brown *et al.* New York: Academic Press. p. 203.

TAMINO, G. & PERETTA, L. (1980) Variations of DNA physico-chemical parameters in its interactions with mutagenic and/or carcinogenic compounds. In *Developments in Biophysical Research*. Ed. Borsellino *et al.* New York: Plenum Press. p. 335.

TAMINO, G., PERETTA, L. & LEVIS, A. G. (1981) Effects of trivalent and hexavalent chromium on physico-chemical properties of mammalian cell nucleic acids and synthetic polynucleotides. *Chem. Biol. Interact.* (in press).

TKESHELASHVILI, L. K., SHEARMAN, C. W., ZAKOUR, R. A., KOPLITZ, R. M. & LOEB, L. A. (1980) Effects of arsenic, selenium and chromium on the fidelity of DNA synthesis. *Cancer Res.*, **40**, 2455.

TSUDA, H. & KATO, K. (1977) Chromosomal aberrations and morphological transformation in hamster embryonic cells treated with potassium dichromate *in vitro*. *Mutat. Res.*, **46**, 87.

UMEDA, M. & NISHIMURA, M. (1979) Inducibility of chromosomal aberrations by metal compounds in cultured mammalian cells. *Mutat. Res.*, **67**, 221.

VALLÉE, M. (1969) Le systeme de transport de sulfate chez *Chlorella pyrenoidosa* et sa régulation. IV. Etudes avec l'ion chromate. *Biochim. Biophys. Acta*, **173**, 486.

VENITT, S. & LEVY, L. S. (1974) Mutagenicity of chromate in bacteria and its relevance to chromate carcinogenesis. *Nature*, **250**, 493.

WHITE, L. R., JAKOBSEN, K. & ØSTGAARD, K. (1979) Comparative toxicity studies of chromium-rich welding fumes and chromium on an established human cell line. *Environm. Res.*, **20**, 366.

WHITING, R. F., STICH, H. F. & KOROPATNICK, D. J. (1979) DNA damage and DNA repair in cultured human cells exposed to chromate. *Chem. Biol. Interact.*, **26**, 267.

WILD, D. (1978) Cytogenetic effects in the mouse of 17 chemical mutagens and carcinogens evaluated by the micronucleus test. *Mutat. Res.*, **56**, 319.